# Gambling, trauma, and the mind: a network analysis of online gambling and personal well-being

**DOI:** 10.1186/s40359-025-03516-z

**Published:** 2025-11-05

**Authors:** Jakub Helvich, Lukas Novak, Zednek Meier, Peter Tavel

**Affiliations:** 1 Olomouc University Social Health Institute, Palacky University in Olomouc, Olomouc, Czech Republic; 2 Department of Pedagogy and Psychology, Faculty of Education, University of Hradec Kralove, Hradec Kralove, Czech Republic

**Keywords:** Online gambling, Online Gambling Disorder Questionnaire, Substance use, Distress, Suicidal ideation

## Abstract

**Background:**

Online gambling has become a significant public health concern due to its accessibility and potential negative impact on personal well-being. However, the complex relationships between online gambling and related factors remain insufficiently explored.

**Objective:**

Therefore, the aim of this study was to investigate the relationships between online gambling disorder (OGD), psychological distress, suicidal ideation, and substance abuse. Additionally, the study examined the influence of adverse childhood experiences (ACEs) on these relationships.

**Methodology:**

Network analyses were conducted using a large nationwide sample of Czech adults (*n* = 3399). Measured variables included OGD, psychological distress (depression, anxiety, and stress scales), suicidal ideation, and substance use behaviors. Separate networks were estimated for males and females. The effect of ACEs on the relationships within the networks was evaluated via the Network Comparison Test (NCT) and edge weight difference tests.

**Results:**

No strong associations between OGD and psychological distress or suicidal ideation were found. Additionally, only weak links were observed between OGD and selected substance use items. Changes in node connections were detected when ACEs were introduced into the networks. Furthermore, the NCT revealed isolated structural and connectivity differences between male and female networks.

**Conclusion:**

While no direct influence of ACEs on the links between OGD and psychological distress was observed, gender-specific differences emerged in how individual OGD items are interconnected. This underscores the need for further research to explore the potential mechanisms through which OGD manifests differently in men and women.

**Supplementary Information:**

The online version contains supplementary material available at 10.1186/s40359-025-03516-z.

## Introduction

Over the past decade, online gambling has rapidly become a critical global public health concern, driven by unprecedented accessibility of internet-based gambling platforms. The rise of smartphones, improved internet access, and the proliferation of digital platforms have transformed gambling into a pervasive activity, attracting individuals who might otherwise avoid offline gambling [45, 46, 62]. Disordered online gambling is defined as a persistent and recurrent pattern of internet-based gambling that causes significant distress and functional impairment (ICD-11: 6C50.1). Recognized as a behavioral addiction, gambling disorder is classified in both the DSM-5 and ICD-11 as a mental health condition [3, 54], with the latter introducing “predominant online gambling” as a specific subtype [34]. This highlights the unique risks tied to internet-based gambling, such as convenience, round-the-clock availability, and escalation of disordered behaviors [45, 46, 62].

Global trends underscore the alarming growth of online gambling engagement. In the United States, the percentage of adults gambling online more than doubled from 6% in 2014 to 13% in 2020 [90], while Canada reported an increase from 2.2% in 2012 to 4.5% in 2018 [23]. In Australia, online gambling participation soared from 0.6% in the late 1990 s to 17.5% in 2019 [58–60]. Numerous European countries have also reported substantial growth in online gambling [19, 26, 29, 100]. In the Czech Republic, 44–56% of individuals aged 15 and over reported some form of gambling in the preceding year, and 17–18% engaged in online gambling [91]. These rising figures are particularly concerning, as online gambling can have far-reaching negative consequences for individual health.

Online gambling has been consistently associated with a wide range of psychological, physical, and behavioral health risks. Individuals with disordered online gambling behaviors often report elevated levels of stress, anxiety, and depression [7, 10, 20, 102]. Studies also indicate that individuals who frequently engage in online gambling experience broader psychosocial harms, including difficulties at work or school [88, 98], reduced engagement in hobbies and social interactions, and less time spent with family and significant others [4, 87]. Online gambling is also closely tied to a cluster of risk behaviors, particularly alcohol consumption, but also smoking, illicit substance use, and other impulsive risk-taking activities [24, 35, 48, 102]. Despite these findings, significant research gaps remain, with many aspects of these relationships inadequately explored.

Suicidal ideation has been repeatedly associated with gambling disorders, largely due to the financial strain, social isolation, and psychological stress that often accompany compulsive gambling [73, 80, 116]. Online gambling disorder also frequently co-occurs with substance abuse [24, 35, 48, 102], further impacting mental health and increasing suicidal ideation [6, 79, 95, 101]. Crucially, it remains unclear how past and present adversities influence susceptibility to online gambling disorder and its mental health implications. Adverse childhood experiences have consistently been linked to multiple high-risk behaviors in adulthood, including substance abuse and decreased mental well-being [25, 33, 63]. However, little research has been conducted on how this factor specifically compounds in the context of online gambling disorder.

Trauma can be broadly defined as exposure to extremely stressful or life-threatening events that overwhelm an individual’s ability to cope [30]. Correspondingly, adverse childhood experiences (ACEs) refer to various forms of abuse, neglect, and household dysfunction occurring before age 18 that can have lasting traumatic impacts on development [16]. ACEs are commonly measured via standardized questionnaires, such as the Adverse Childhood Experiences International Questionnaire (ACE-IQ), developed by the WHO [123]. The ACE-IQ reliably captures a broad range of early adversities (e.g., physical, emotional, and sexual abuse,neglect; domestic violence; household substance abuse; or mental illness [123]. To understand how ACEs might alter connections among gambling, distress, and substance use, we draw on trauma-informed addiction models and theories of emotion dysregulation. In the context of the present study, these include the allostatic-load or stress-vulnerability model [96, 106] and the self-medication hypothesis [68, 114]. Emotion-dysregulation frameworks further encompass the process model of emotion regulation [52] and the biosocial theory of emotion dysregulation [103]. Research demonstrates that early adversity can dysregulate stress response systems and promote maladaptive coping strategies [5, 66, 112]. According to these frameworks, individuals with a history of ACEs may develop: (1) increased emotional reactivity and emotion dysregulation, leading to greater vulnerability to psychological distress [5, 112],(2 impaired impulse control that facilitates substance use and gambling behaviors [5, 32], and (3) a tendency to use these behaviors as affect regulation strategies to escape trauma-related distress [5, 112].

Previous studies on online gambling have primarily relied on traditional statistical methods, such as univariate regression analysis, which often fail to capture bidirectional relationships and the broader systemic patterns associated with online gambling [13, 92]. To address these limitations, network analysis offers a more advanced method for understanding the multifaceted nature of online gambling disorder within and across the broader spectrum of related symptoms. This relatively recent approach investigates complex systems by analyzing structures and patterns in data, drawing on mathematical principles such as graph theory and network optimization [15]. Network analysis enables researchers to identify key nodes—important behaviors and psychological traits—and the pathways that connect them, revealing how specific factors contribute to the development of online gambling behaviors. By applying network analysis, researchers can gain deeper insights into the interconnections among online gambling symptoms and their links to personal well-being, clarifying how specific factors influence these relationships at the item-to-item level [57].

Therefore, the first objective of this study is to address existing research gaps by examining the relationships among online gambling disorder items and their associations with psychological distress, suicidal ideation, and substance abuse using network analysis. The second objective is to investigate how adverse childhood experiences (ACEs) influence the relationships between online gambling disorder, psychological distress, and substance abuse.

## Methods

### Participants and procedure

Data collection for the study was conducted in the Czech Republic between October and December 2024 through an online questionnaire administered by the Olomouc University Social Health Institute (OUSHI). The survey employed convenience and snowball sampling methods to recruit participants. Participation was entirely voluntary, and respondents could withdraw from the survey at any time. Informed consent was obtained at the beginning of the questionnaire. The study received ethical approval from the Ethics Board of the Faculty of Theology at Palacký University (Approval No. 2020/4) and was conducted in accordance with the ethical requirements of the Convention on Human Rights and Biomedicine (40/2000 Coll.).

A total of *n* = 4,810 participants completed the survey. To ensure data integrity, multiple quality-screening procedures were implemented. First, respondents who did not provide sociodemographic information and therefore did not begin completing the questionnaires were excluded (*n* = 350). Second, respondents younger than 18 years of age were excluded. Third, participants whose responses to repeated demographic control questions (weight, height, and age) deviated beyond predefined tolerance thresholds (± 2 kg, ± 2 cm, ± 1 year) were removed (*n* = 144). These demographic checks were presented both at the beginning and end of the questionnaire to detect and eliminate inconsistencies. Fourth, participants whose survey completion times fell below an empirically established threshold of 18 min—determined through pilot testing—were excluded (*n* = 683). Fifth, only respondents identifying as Czech nationals were retained, resulting in the exclusion of *n* = 157 non-Czech participants.

Sixth, to prevent multiple submissions by the same individuals, an examination involving calculating the probability of browser type and version matches across submissions was done using the following equations:

$$p=\frac{{\sum }_{{i}_{=1}}^{n}{x}_{ji}\ne 0}{n}$$

In this equation, *p* represents the probability of a match between the browser type and version for each respondent. It is calculated by identifying the number of rows where the i-th element from the interviewer's code column corresponds to the j-th element in the browser type and version row used to complete the questionnaire. The variable *n* denotes the total number of rows in the matrix. Based on these match probabilities, a tolerance threshold was subsequently determined:

$$\begin{aligned} tolerance\,limit=&\overline{p}*med\left({m}_{1},{m}_{2},{m}_{3}\dots {m}_{k}\right)\\&*\sum_{q=1}^{n}\left({q}_{m}>1\right)\end{aligned}$$

In this formula, *m* represents the number of questionnaires completed by a single respondent, and *q* identifies the respondent. Nevertheless, no attempts of submitting multiple entries were identified in the dataset.

Outliers were assessed using the Median Absolute Deviation (MAD) method applied to the study scales. Thirty-four participants were flagged as potential outliers; however, further examination revealed no systematic evidence of low-quality responses, and these cases were therefore retained. The final analytic sample consisted of *n* = 3,399 participants (Age: M = 27.4, SD = 12.3, range = 66; 61.46% women).

### Measures

#### Online gambling disorder questionnaire (OGD-Q)

The Online Gambling Disorder Questionnaire (OGD-Q) is a self-report instrument designed to assess the severity of disordered online gambling behaviors based on established diagnostic criteria [49]. The questionnaire comprises 11 items that evaluate core dimensions of online gambling disorder, such as the tendency to increase wager amounts to maintain excitement (e.g., *"Do you feel the need to spend more and more money to achieve the high you desire?"*). Each item is rated on a five-point Likert scale ranging from 1 (*never*) to 5 (*every day*. In the original study, the OGD-Q demonstrated good psychometric properties, with factor loadings between 0.63 and 0.85 and high internal consistency (Cronbach’s α > 0.94; [49]). Total scores are calculated by summing responses across all 11 items, yielding a possible range of 11 to 55, where higher scores reflect greater severity of disordered online gambling. In this study, we utilized the Czech version of the scale, validated by [55]. The internal consistency was good: Cronbach’s α = 0.89, 95% CI[0.88–0.9] and McDonald's ω: 0.92.

#### Overall depression severity and impairment scale (ODSIS)

The Overall Depression Severity and Impairment Scale (ODSIS) is a self-report instrument designed to assess both the severity of depressive symptoms and their impact on daily functioning [11]. It includes five items that evaluate the frequency of depressive distress and the extent to which these symptoms interfere with everyday activities, including work and social interactions [71]. Each item is rated on a five-point scale, yielding a total score from 0 to 20, with higher scores reflecting greater depressive symptomatology. The ODSIS has demonstrated strong psychometric properties in both clinical and non-clinical populations [71, 83]. In this study, we employed the validated Czech adaptation of the abbreviated ODSIS developed by Sandora et al. [110]. In the present sample, the scale showed excellent internal consistency (Cronbach’s α = 0.95, 95% CI [0.94, 0.95]; McDonald's ω = 0.96).

#### Overall anxiety severity and impairment scale (OASIS)

The Overall Anxiety Severity and Impairment Scale (OASIS) is a brief self-report measure designed to assess the frequency, intensity, and functional impact of anxiety symptoms [93]. It consists of five items that evaluate how often individuals experience anxiety-related distress and the extent to which these symptoms interfere with daily activities such as work, social interactions, and personal responsibilities. Each item is rated on a five-point scale from *0 (never)* to *4 (all the time)*, yielding a total score between 0 and 20, with higher scores indicating greater anxiety severity. The OASIS has demonstrated strong psychometric properties, including excellent internal consistency, test–retest reliability, and convergent validity with other established anxiety measures [21, 22, 84, 93]. In this study, we used the validated Czech version by Sandora et al. [110]. Internal consistency in the present sample was good (Cronbach’s α = 0.89, 95% CI [0.88, 0.90]; McDonald's ω = 0.92).

#### Depression anxiety stress scale (DASS-21)

The Depression Anxiety Stress Scale (DASS) is a widely used instrument designed to assess three interrelated emotional states: depression, anxiety, and stress [77]. The short-form version, DASS-21, consists of 21 items divided into three subscales—Depression, Anxiety, and Stress—each comprising seven items. While each subscale targets a specific emotional domain, together they provide a comprehensive assessment of broader negative affectivity. For this study, only the Stress subscale was used. Participants rated how frequently each statement applied to them over the past week on a four-point Likert scale (*0* = *not at all, 1* = *sometimes, 2* = *often, 3* = *almost always*). Higher scores reflect greater stress. This study employed the validated Czech version of the DASS-21 [120]. In the present sample, the Stress subscale showed good internal consistency (Cronbach’s α = 0.84, 95% CI [0.83, 0.85]; McDonald’s ω = 0.90.

#### Suicidal ideation attributes scale (SIDAS)

The Suicidal Ideation Attributes Scale (SIDAS) is a self-report instrument developed to assess the severity and attributes of suicidal ideation over the past month. It consists of five items addressing key dimensions of suicidal thoughts, including frequency, controllability, closeness to attempting suicide, distress caused, and impact on daily functioning [119]. Each item is rated on an 11-point scale ranging from *0 (not at all)* to *10 (always/very much)*, yielding a total score from 0 to 50. Higher scores reflect greater severity of suicidal ideation. The SIDAS has demonstrated strong psychometric properties, including high internal consistency (Cronbach’s α = 0.91) and good construct validity. For this study, we employed the validated Czech version of the SIDAS [99]. In the present sample, the SIDAS showed good internal consistency (Cronbach’s α = 0.77, 95% CI [0.75, 0.78]; McDonald’s ω = 0.89.

#### Adverse Childhood Experience International Questionnaire (ACE-IQ)

The Adverse Childhood Experiences International Questionnaire (ACE-IQ) is a 43-item self-report screening measure designed to assess exposure to traumatic events during childhood [123]. The questions are grouped into 13 categories, covering various types of abuse, neglect, and household-related adverse experiences. For the purposes of this study, only family-related adverse experiences were included. Respondents rated items on 5-point, 4-point, or dichotomous scales (*0* = *No, 1* = *Yes*. The ACE-IQ total score was calculated using the more conservative frequency-based scoring method, resulting in scores ranging from 0 to 13, with higher scores indicating greater exposure to childhood adversity. For this study, the Czech version of the ACE-IQ was developed using a back-translation procedure. In the present sample, the ACE-IQ demonstrated good internal consistency (Cronbach’s α = 0.80, 95% CI [0.79, 0.81]; McDonald’s ω = 0.86.

#### Health risk behaviours

Risk behaviors in this study were assessed using five items addressing substance-related risk-taking. Participants were asked: *“How often in the past month have you…”* followed by the questions: (1) used tobacco products (e.g., cigarettes, e-cigarettes, chewing tobacco, snuff, pipes, cigars); (2) consumed alcohol; (3) used cannabis (e.g., marijuana, hashish); (4) used non-cannabis illicit drugs (e.g., ecstasy, cocaine, methamphetamine, LSD, heroin, or others); and (5) taken a prescription drug not prescribed to you by a doctor. Responses were rated on a six-point Likert scale from 1 (*Never*) to 6 (*Many times a day*), with higher scores indicating more frequent engagement in these behaviors.

#### Data analysis

Network analysis was used to explore and assess associations between the variables in the dataset. To conduct the analysis, Markov Random Fields (MRFs) was employed. MRFs are grounded in graph theory, with nodes (circles) representing variables and edges (connections between nodes) representing pairwise associations while conditioning on all other variables. MRFs were chosen due to several advantages over traditional statistical methods. First, they estimate each edge in the network while simultaneously controlling for the effects of all other variables [39]. This allows adjustment for specific variables within the network. Second, MRFs facilitate the visualization of complex interconnections among variables, enabling the identification of reciprocal relationships, such as feedback loops—for example, cases where variable A influences variable B, which in turn feeds back to affect variable A [28]. Third, MRFs allow examination of relationships at the level of individual items rather than aggregated scales, providing a more granular view of the data [41].

Network estimation was conducted using the qgraph package in R [37], based on polychoric correlations for ordinal data and the EBICglasso estimator [44, 117], with Least Absolute Shrinkage and Selection Operator (LASSO) regularization applied to minimize spurious edges. LASSO penalization was controlled by the tuning parameter (γ), which was set conservatively at 0.5. Prior research indicates that this threshold effectively reduces false positives while retaining meaningful connections within the network [64, 75]. The network layout was generated using the Fruchterman–Reingold algorithm, which arranges nodes in a visually interpretable structure by balancing attraction and repulsion forces. Following Christensen and Golino [27], effect-size guidelines for edge weights were interpreted as 0.15 (weak), 0.25 (moderate), and 0.35 (strong), consistent with thresholds reported in previous studies [47, 56].

Centrality parameters were examined to assess the relative importance of individual nodes within the network. Specifically, four centrality indices were computed: (1) strength centrality, defined as the sum of the absolute edge weights connected to a node, reflecting its overall connectivity; (2) expected influence, calculated as the sum of edge weights (including sign), which captures both positive and negative associations and highlights nodes with broad activating or inhibiting potential; (3) betweenness centrality, indicating how often a node lies on the shortest path between two other nodes, thus identifying potential bridge symptoms; and (4) closeness centrality, representing the average inverse distance from a node to all others, reflecting how efficiently information can spread through that node. These indices help to identify nodes that may function as core symptoms or key pathways, thereby enhancing understanding of the interrelations between online gambling disorder, psychological distress, and associated behaviors.

Node predictability, which indicates how well a given variable (node) can be estimated based on its connections to other variables, was calculated. Additionally, a non-parametric bootstrap with 1,000 samples (with replacement) was performed to assess the strength of edge weights (connections between variables) using the bootnet package in R [36]. The stability of network estimates, including edge weights and their confidence intervals, was assessed through a case-dropping bootstrap with 1,000 subsamples, examining how consistently central nodes remained dominant when portions of the data were removed.

Prior to formal statistical network comparisons, a visual inspection was conducted to identify observable differences between networks. Network Comparison Tests (NCTs) were then used to evaluate: (1) how the inclusion of ACEs altered the connections between nodes and which nodes were most affected, and (2) whether ACEs influenced network connections differently in males and females. To further examine the effect of ACEs on associations between individual nodes, an ordered edge-weight difference matrix was generated. This matrix displayed all possible node pairs and quantified the extent to which associations between them changed after ACEs were introduced into the network. This was done by subtracting the edge values of the network without ACEs from the corresponding edges in the ACE-included network.

To compare networks between males and females, the R package NetworkComparisonTest [118] was employed. Based on 1,000 data-driven permutations, we tested for network structure invariance (differences in edge weights) and global strength invariance (differences in the absolute sum of edge weights). Additional R packages used in the analyses included psych [104], lavaan [109], psychtoolbox [94], bootnet [40], MissMech [65], and psychonetrics [38].

## Results

The assumption of multivariate normality was evaluated using Mardia’s tests for skewness (651.74) and kurtosis (2956.65), both of which indicated significant deviations from normality. Examination of residual plots and Breusch–Pagan statistics further suggested potential heteroscedasticity. Given these violations, Spearman’s rank correlations and other non-parametric methods were used to examine associations between variables. Missing data patterns were assessed with Little’s MCAR test, which indicated no systematic pattern of missingness; therefore, missing data were handled using listwise deletion.

### Descriptive statistics

Based on the sociodemographic data presented in Table 1, the final sample consisted of *n* = 3,399 adult respondents (Age: M = 27.4, SD = 12.3, range = 66). The majority of participants were female (61.46%), and 38.54% were male. In terms of economic status, most respondents were students (57.37%), followed by employed individuals (32.48%). Regarding family status, more than half reported not being in a relationship (55.93%), about a quarter indicated being in a relationship or partnership (24.62%), and the remainder were married (16.12%), divorced (2.85%), or widowed (0.47%).

**Table 1:** sociodemographic characteristics of the sample (*n* = 3399)

Variable	n (%)
Gender	
Female	2089 (61.46)
Male	1310 (38.54)
Economic status	
Student	1950 (57.37)
Employed	1104 (32.48)
Self-Employed	173 (5.09)
Unemployed	64 (1.88)
Retired	64 (1.88)
On maternity/parental leave	44 (1.29)
Income (in CZK)	
under 20.000	129 (3.81)
21 000—30.000	325 (9.58)
31.000—40.000	447 (13.17)
41.000—50.000	544 (16.03)
51.000—60.000	543 (16.00)
61.000—70.000	414 (12.20)
71.000—80.000	373 (10.99)
81.000—90.000	209 (6.16)
91.000—100.000	169 (4.98)
over 101.000	246 (7.25)
Education	
Elementary	272 (8)
Vocational school	280 (8.24)
Secondary school	2172 (63.9)
Higher vocational school	94 (2.77)
Bachelor's degree (B.A.)	340 (10)
Master's degree (M.A. or PhD)	241 (7.09)
Family status	
Not in a relationship	1901 (55.93)
Married	548 (16.12)
Divorced	97 (2.85)
Widower/Widow	16 (0.47)
In a relationship/partnership	837 (24.62)

### Network analysis

The overall unadjusted network for the full sample (Supplementary Material 1) revealed positive direct links among anxiety, stress, depression, and suicidal ideation (*r* = 0.21–0.48), with no strong associations between OGD-Q items and psychological distress or suicidal ideation. Additionally, no strong or weak connections were observed between OGD-Q items and substance use items, nor were significant links detected between substance use items and psychological distress or suicidal ideation. Controlling for ACEs (Supplementary Material 2) did not alter edge strengths, although some connections emerged between ACEs and both psychological distress and suicidal ideation. Sex-segmented networks for males (Supplementary Material 3) and females (Supplementary Material 4) were visually inspected, and only minimal differences were observed. Edge weights between OGD-Q items and psychological distress or suicidal ideation, as well as between OGD-Q items and substance use items, increased slightly in strength but remained insignificant in both networks. Incorporating ACEs into sex-differentiated networks (Supplementary Materials 5 and 6) produced no noticeable changes in edge weights. Since no visual differences were observed, no statistical network comparison tests were conducted.

**Fig. 1:**
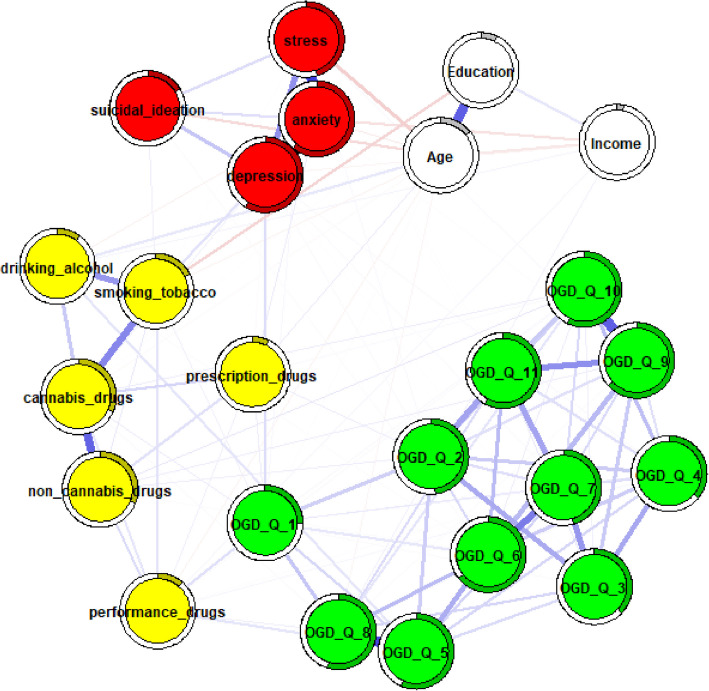
Full sample network adjusted for age,education, and income. Note. Thicker edges represent stronger associations, while thin edges reflect weak associations. Blue edges reflect positive associations, while red edges represent negative associations. Rings around the circles refer to node predictability. A higher degree of gray color inside these circles reflects higher node predictability. OGD-Q_1—11 = Online gambling disorder questionnaire items, depression = ODSIS total score, anxiety = OASIS total score, stress = DASS21 stress subscale total score, suicidal ideation = SIDAS total score, yellow nodes represent individual substance use items

In the next step, networks adjusted for age, education, and income were estimated and compared. To control for potential confounding, nodes representing these variables were integrated into the networks, allowing for clearer interpretation of conditional dependencies both among the primary study variables and between the confounders and other variables in the network. The adjusted full-sample network (Fig. 1) revealed no strong associations between OGD-Q items and substance use behaviors. Similarly, no strong edges were observed between online gambling disorder items and psychological distress or suicidal ideation.

**Fig. 2:**
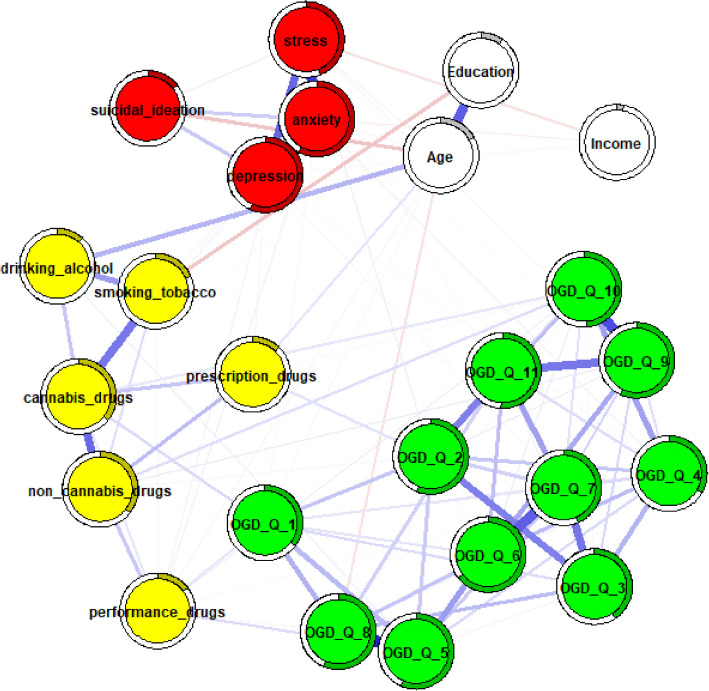
male network adjusted for age, education, and income

**Fig. 3:**
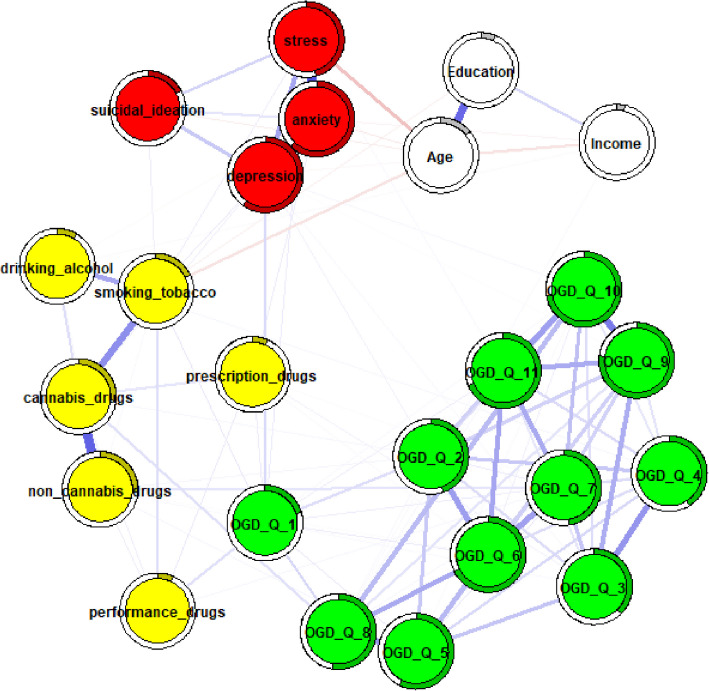
female network adjusted for age, education, and income. Note. Thicker edges represent stronger associations, while thin edges reflect weak associations. Blue edges reflect positive associations, while red edges represent negative associations. Rings around the circles refer to node predictability. A higher degree of gray color inside these circles reflects higher node predictability. OGD-Q_1—11 = Online gambling disorder questionnaire items, depression = ODSIS total score, anxiety = OASIS total score, stress = DASS21 stress subscale total score, suicidal ideation = SIDAS total score, yellow nodes represent individual substance use items

Several differences in edge strength were observed between unadjusted and adjusted networks for males (Fig. 2) and females (Fig. 3), as well as between the adjusted networks themselves, particularly among OGD-Q items. First, some edges changed from non-significant to weak (males: OGD-Q_5 – OGD-Q_6, *r* = 0.16; OGD-Q_7 – OGD-Q_11, *r* = 0.16; females: OGD-Q_3 – OGD-Q_9, *r* = 0.16). Second, several edges increased in strength from weak to moderate (males: OGD-Q_6 – OGD-Q_7, *r* = 0.27; OGD-Q_9 – OGD-Q_11, *r* = 0.25) or from moderate to strong (males: OGD-Q_5 – OGD-Q_8, *r* = 0.36). These changes were particularly noticeable in the male networks. Additionally, OGD-Q_1 (“Do you feel the need to spend more and more money to get the high you desire?”) remained a main bridging node between online gambling disorder and some substance use items after adjustment, although these connections were weak or non-significant. Finally, no strong connections were observed between OGD-Q items and psychological distress in either adjusted network.

### Effect of ACEs on the relationships in the networks

**Fig. 4:**
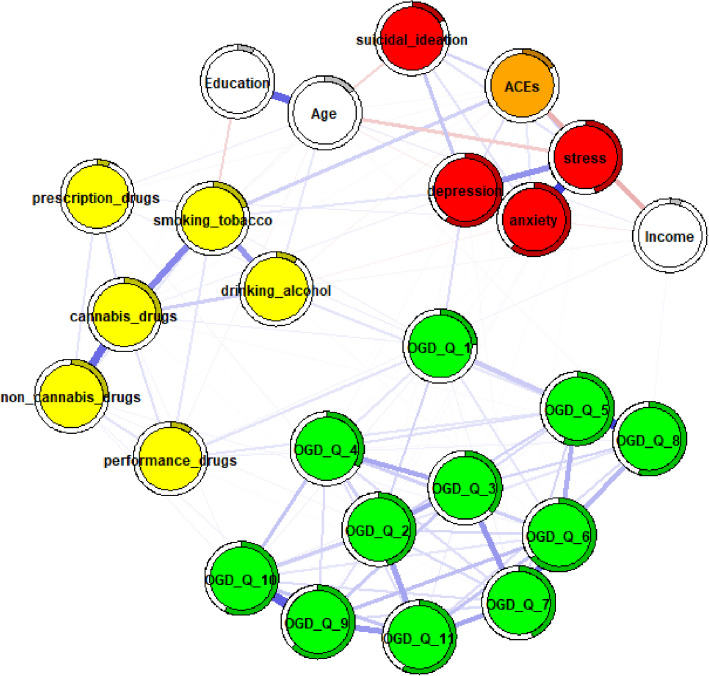
full sample ACEs network adjusted for age, education, and income. Note. Thicker edges represent stronger associations, while thin edges reflect weak associations. Blue edges reflect positive associations, while red edges represent negative associations. Rings around the circles refer to node predictability. A higher degree of gray color inside these circles reflects higher node predictability. OGD-Q_1—11 = Online gambling disorder questionnaire items, depression = ODSIS total score, anxiety = OASIS total score, stress = DASS21 stress subscale total score, suicidal ideation = SIDAS total score, ACEs = ACE-IQ total score, yellow nodes represent individual substance use items

**Fig. 5:**
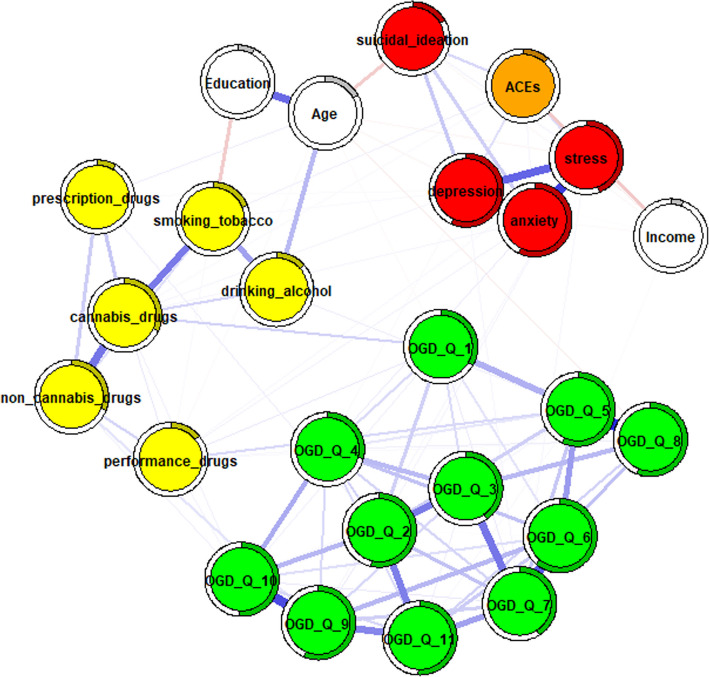
male ACEs network adjusted for age, education, and income

**Fig. 6:**
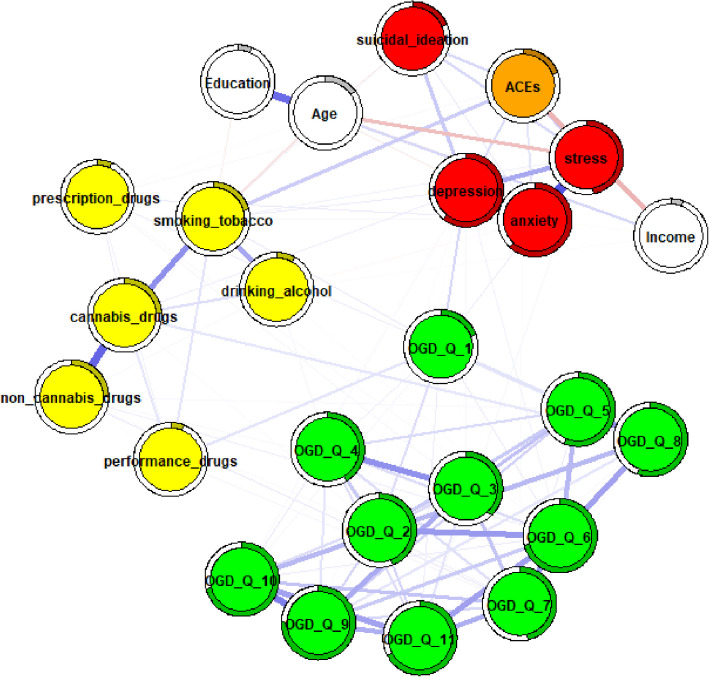
female ACEs network adjusted for age, education, and income. Note. Thicker edges represent stronger associations, while thin edges reflect weak associations. Blue edges reflect positive associations. Rings around the circles refer to node predictability, while red edges represent negative associations. A higher degree of gray color inside these circles reflects higher node predictability. OGD-Q_1—11 = Online gambling disorder questionnaire items, depression = ODSIS total score, anxiety = OASIS total score, stress = DASS21 stress subscale total score, suicidal ideation = SIDAS total score, ACEs = ACE-IQ total score, yellow nodes represent individual substance use items

When the ACEs total score was introduced into the adjusted networks (Figs 4, 5 and 6), selective modulations were identified, as indicated by ordered edge-weight difference matrices (Supplementary Materials 7–9), particularly among OGD-Q items in the female network, while the interconnections among core mental health scores remained largely stable.

In the male adjusted network, increases in edge weights were observed between OGD-Q_2–OGD-Q_7 (+ 0.0236) and OGD-Q_1–OGD-Q_10 (+ 0.0234), while decreases were found between OGD-Q_9–OGD-Q_11 (–0.0287), OGD-Q_6–OGD-Q_9 (–0.0263), and OGD-Q_4–OGD-Q_5 (–0.0253). A more notable change included a decrease in the edge weight between OGD-Q_10 and non-cannabis drug use (–0.027).

In the female adjusted network, several modulations were observed, including increases in edge weights such as OGD-Q_6–OGD-Q_8 (+ 0.0167), OGD-Q_6–OGD-Q_9 (+ 0.0128), and OGD-Q_5–OGD-Q_8 (+ 0.0110). Conversely, negative changes were detected in edges such as OGD-Q_1–OGD-Q_8 (–0.0236) and OGD-Q_9–OGD-Q_10 (–0.0105). Additionally, an increase was found between OGD-Q_6 and non-cannabis drug use (+ 0.028).

In the full-sample adjusted network, ACEs predominantly altered the OGD-Q item cluster and connections between OGD-Q items and certain substance use behaviors. Notable changes included an increase between OGD-Q_6–OGD-Q_10 (+ 0.0174) and a decrease between OGD-Q_7–OGD-Q_9 (–0.0213). Furthermore, the connection between OGD-Q_10 and non-cannabis drug use decreased (–0.0163).

Overall, across all adjusted networks, the edges involving OGD-Q items and substance use behaviors showed the most frequent changes with the inclusion of ACEs, whereas the interconnections among mental health scores remained unaffected.

### Network comparison testing

A network comparison test was conducted to examine differences in structure and overall connectivity between the adjusted male and female networks when the ACEs total score was included. The network invariance test revealed only a small, non-significant difference in network structure between groups (*M* = 0.199, *p* = 0.484), indicating that the configuration of connections (i.e., the arrangement and strength of edges) differed only slightly. Similarly, the global strength invariance test showed no significant difference in overall connectivity, defined as the sum of absolute edge weights, with global strength values of 8.31 for one group and 8.80 for the other (*S* = 0.489, *p* = 0.560). Summary statistics of edge-weight differences indicated a range from –0.200 to + 0.146, with a median of 0 and a mean near 0 (0.0009). Edges with negative differences (approaching –0.200) suggest slightly stronger connections in the male network, whereas edges with positive differences (up to + 0.146) suggest stronger connections in the female network. Overall, while most connections were similar across groups, a subset of edges displayed localized differences, contributing to the minor dissimilarities in network structure and connectivity.

Overall, these findings indicate only minor global differences in network structure and connectivity between groups, although some specific edges differed in strength. This underscores slight heterogeneity in network alterations between males and females when adjusted for age, education, and income. A complete edge-weight difference matrix is provided in Supplementary Material 10, and Supplementary Material 11 presents a graphical visualization of key numerical differences between nodes in the network.

### Network accuracy and stability

All bootstrap results for both adjusted and unadjusted networks, along with the centrality plot estimates, are provided in Supplementary Materials 12–47. For easier navigation, see Table 2.

**Table 2:** sorted supplementary materials for all estimated networks

Estimated network	Strength centrality estimates	Accuracy of edge weight estimates	bootstrapped difference test of node strength
Unadjusted full sample network	Supplementary_material_12	Supplementary_material_24	Supplementary_material_25
Unadjusted male network	Supplementary_material_13	Supplementary_material_26	Supplementary_material_27
Unadjusted female network	Supplementary_material_14	Supplementary_material_28	Supplementary_material_29
Unadjusted full sample network with ACEs	Supplementary_material_15	Supplementary_material_30	Supplementary_material_31
Unadjusted male network with ACEs	Supplementary_material_16	Supplementary_material_32	Supplementary_material_33
Unadjusted female network with ACEs	Supplementary_material_17	Supplementary_material_34	Supplementary_material_35
Adjusted full sample network	Supplementary_material_18	Supplementary_material_36	Supplementary_material_37
Adjusted male network	Supplementary_material_19	Supplementary_material_38	Supplementary_material_39
Adjusted female network	Supplementary_material_20	Supplementary_material_40	Supplementary_material_41
Adjusted full sample network with ACEs	Supplementary_material_21	Supplementary_material_42	Supplementary_material_43
Adjusted male network with ACEs	Supplementary_material_22	Supplementary_material_44	Supplementary_material_45
Adjusted female network with ACEs	Supplementary_material_23	Supplementary_material_46	Supplementary_material_47

## Discussion

The objective of this study was to examine the relationships between online gambling disorder, psychological distress, suicidal ideation, and substance abuse, as well as to assess how adverse childhood experiences (ACEs) influence these associations. The results revealed no strong associations between OGD-Q items and psychological distress or suicidal ideation. Additionally, several moderate to strong positive edges between OGD-Q items were detected in both adjusted female and male networks. Moreover, ACEs influenced the strength of connections, particularly between OGD-Q items and between OGD-Q items and substance use items. However, no significant influence of ACEs was observed on the associations between OGD-Q items and psychological distress or suicidal ideation. Finally, the network comparison test revealed only marginal differences between the adjusted male and female networks, primarily involving OGD-Q items.

Multiple lines of evidence indicate that individuals experiencing gambling disorder often exhibit elevated rates of mental health issues and suicidality. Prior research has documented that the severity of gambling behaviors correlates with higher levels of psychological distress and increased self-harm risk [72, 73, 76, 80, 111]. However, research on online gambling disorder remains relatively limited compared to offline gambling–related psychological distress and self-harm risks [88]. Additionally, when examined in multivariate or network contexts, direct connections often appear weaker than suggested by bivariate analyses [107]. One possible explanation is that the association between gambling and psychological distress is indirect or mediated by other variables. For example, Sundqvist and Wennberg [115] found that although frequent gamblers had about twice the odds of suicidal ideation compared to non-gamblers, when controlling for lifetime mental health disorders, the link between gambling disorder and suicide attempts was no longer significant. The most significant factors for gamblers who attempted suicide were severe depression and heavy drug abuse, rather than gambling severity per se [53, 115]. Similarly, Błoch and Misiak [14] noted that in their comorbidity networks of online gaming, cross-domain edges were generally weak, making it difficult to pinpoint direct symptom-to-symptom connections between conditions. This suggests that treating gambling disorder in isolation may not resolve distress or suicide risk unless co-occurring depression and related consequences are also addressed. At the same time, it is important to recognize that network approaches are correlational in nature and do not allow for causal inference or determination of directionality. Nevertheless, understanding these indirect links underscores the importance of examining how early-life adversity may shape gambling-related risk behaviors across demographic groups.

Although ACEs did not significantly alter unadjusted network structures, controlling for age, education, and income revealed pathways linking early adversity to gambling and substance use behaviors. This suggests that sociodemographic factors shape how ACEs affect these relationships, particularly between OGD-Q items and between OGD-Q items and substance use. For example, younger individuals or those with lower educational attainment may adopt distinct coping strategies in response to childhood adversity, which may strengthen or alter pathways linking ACEs to risky behaviors such as gambling or substance use compared to older or more educated counterparts. This interpretation is supported by prior studies showing that the effects of ACEs often become clearer or stronger when age-related and socioeconomic factors are considered [1, 12, 122]. Similarly, educational attainment can mitigate or exacerbate the psychological impacts of early-life stressors, influencing behavioral patterns and susceptibility to addictive behaviors [21, 22, 70, 85]. Therefore, it is essential to account for sociodemographic factors in research on behavioral and mental health to accurately estimate the impact of childhood adversity. These findings also highlight the importance of examining how OGD co-occurs with substance misuse across diverse populations.

When ACEs were introduced into the adjusted networks, selective modulations were observed in connections among OGD-Q items, but not between OGD-Q items and substance use behaviors, while the interconnections among core mental health scores remained largely unchanged. This pattern aligns with trauma-informed addiction models [5, 112], which propose that ACEs amplify connections between maladaptive coping behaviors while leaving broader psychological distress networks relatively intact. Specifically, increased emotional reactivity [5, 112] may explain why ACEs strengthened links between certain OGD-Q items and substance use, as both behaviors can serve as dysregulated attempts to manage trauma-related distress. Moreover, impaired impulse control [5, 32] likely underlies the ACEs-mediated association of gambling behaviors with substance use, as both involve reward-seeking despite negative consequences.

Substance abuse commonly co-occurs with frequent gambling, underscoring the need to view these behaviors as interconnected rather than separate clusters [9, 51, 53]. A meta-analysis by Lorains et al. [76] found high rates of alcohol and drug use disorders among frequent gamblers, making substance abuse one of the most frequent comorbidities of gambling disorder. This finding is consistent with more recent network-analytic research by Zarate et al. [126], who examined ten addictive behaviors (including online gambling) and observed positive associations across addictions, with gambling-related symptoms emerging as highly central in the network. Zarate et al. [126] also reported that disordered gambling tendencies had the highest centrality, suggesting that gambling symptoms may act as a bridge that maintains or exacerbates other addictive behaviors, including substance use. Similarly, Błoch and Misiak [14] found that online gambling disorder and substance-related addictive behaviors showed several interconnections, with gambling symptoms often statistically linked to substance use indicators in their network models. Other studies further confirm that, when controlling for relevant factors, results reveal significant positive associations between online gambling disorder and substance abuse in both men and women [97, 111, 124]. Additionally, the emergence of strong connections—particularly negative ones, such as between borrowing money and deception or between borrowing money and experiencing withdrawal-like symptoms when limiting gambling—highlights the complexity of gambling disorder symptoms. These findings suggest the existence of different subtypes or symptom patterns within the disorder and may also point to compensation mechanisms, where engagement in certain behaviors (e.g., borrowing) reduces the likelihood of others (e.g., deception). Nevertheless, further research is needed to clarify the directionality of these associations. Overall, these findings underscore the complexity of gambling disorder’s symptom network, particularly its interplay with substance misuse. Importantly, understanding how these patterns vary by gender may reveal differences in symptom interrelations.

Gender differences in disordered gambling have been well-documented, and recent research using similar methodologies has confirmed that the interrelationships among gambling symptoms can vary between men and women [8, 81, 97, 124]. Previous research also suggests that while the most central symptom was the same for both sexes, the withdrawal criterion (“restlessness/irritability when trying to cut down”), notable differences emerged in the next-most central features [78]. Specifically, men’s gambling networks were characterized by tolerance (“needing to gamble with increasing amounts of money”) as the second most central symptom, whereas women’s networks showed chasing losses (“returning to get even after losses”) as the second most central [78]. In other words, men’s gambling tendencies appear more driven by escalation and excitement, while women’s may be more driven by loss-chasing behaviors, potentially linked to financial strain. This indicates that the structure and emphasis of gambling disorder symptoms vary by gender, reflecting distinct gambling-related experiences [8, 78]. Beyond individual symptom centrality, whole-network comparisons also show significant gender-based differences in the pattern of symptom interconnections. Baggio et al. [8] found that men are more likely to be at-risk or frequent gamblers than women, suggesting that men may develop a broader “full spectrum” of gambling symptoms. Similarly, Błoch and Misiak [14] reported that men were more prone to simultaneous disordered online gambling, whereas women demonstrated slightly different comorbidity patterns. Taken together, these findings support gender-tailored prevention and intervention strategies that account for differences in motivations and comorbidities. In addition to gender, adverse childhood experiences have independently been shown to shape gambling-related trajectories.

A growing body of longitudinal and cross-sectional studies has examined how ACEs contribute to gambling behaviors and their comorbid networks [50, 74, 113]. For instance, Bristow et al. [18] found that exposure to multiple ACEs was associated with higher odds of being an at-risk or frequent gambler. Their results corroborated other findings showing that individuals with histories of childhood maltreatment or household dysfunction are significantly more likely to develop gambling disorders in adulthood [31, 43, 74]. Importantly, ACEs not only increase the risk of gambling disorder but are also associated with greater comorbidity. Childhood adversities have been linked to individuals with co-occurring gambling, mental health, and substance use problems [18, 61]. A study by Kessler et al. [67] suggested that early-life stressors can interact with genetic vulnerabilities to produce a spectrum of addictive behaviors. In practical terms, individuals with traumatic childhoods are more likely to struggle simultaneously with gambling, substance abuse, and psychological distress. The network impact of ACEs has been demonstrated by higher connectivity among these variables. Roberts et al. [105], for example, observed that in individuals with high ACE scores, partial correlations between gambling symptoms and substance use were notably stronger than in those without ACEs, indicating that adversity can strengthen these associations. Conversely, when ACE factors are not considered (as in unadjusted networks), the interplay may be underestimated. This may explain why adding ACEs did not visibly change the unadjusted network structure in the present study, whereas their influence became observable once modeled in adjusted networks. However, the absence of an ACE effect on the relationship between online gambling disorder and psychological distress suggests sample-specific factors, such as variation in severity or the influence of specific confounders. Alternatively, it may reflect that online gambling disorder, although commonly comorbid with distress or suicidal ideation, manifests in unique ways, where different motivations or behaviors could diminish the direct effect of ACEs on these associations. Together, these findings underscore the multilayered relationships between early-life adversity, sociodemographic factors, substance use, and gender, all of which contribute to the heterogeneous presentation of gambling disorders and their comorbidities.

## Implications

### Implications for research

The findings suggest several directions for future research. First, the absence of strong links between OGD and psychological distress or suicidal ideation warrants exploration of alternative pathways through which gambling behaviors may affect mental health. Longitudinal studies are needed to clarify whether gambling disorder precedes or results from psychological distress, substance abuse, or other risk factors. While previous longitudinal studies have provided convincing evidence for offline gambling [2, 69, 125], research on online gambling remains limited [121]. Second, the modest but noteworthy associations between OGD and substance use, particularly non-cannabis drugs and performance-enhancing substances, highlight the need to investigate shared behavioral and neurobiological mechanisms. Future research should also examine whether these associations differ across gambling subtypes and severity levels. Third, the significant influence of adverse childhood experiences (ACEs) on the associations between OGD and substance use underscores the importance of studying early-life risk factors. Longitudinal ACEs data could help determine whether early adversity predisposes individuals to gambling-related substance use while also identifying protective factors that mitigate these risks. Finally, gender-based differences in OGD-related networks call for further investigation into underlying mechanisms, including gender-specific motivations, coping strategies, and social influences. In addition, socioeconomic status, education, and cultural factors should be considered to better understand disparities in gambling disorder trajectories.

### Implications for practice

The findings have important implications for clinical practice, public health, and policy. Given the lack of strong links between OGD and psychological distress or suicidal ideation, interventions should address broader lifestyle, psychological, and contextual factors rather than assuming a direct causal link. Promising approaches include Internet-delivered, therapist-assisted CBT protocols that reduce gambling severity and psychiatric symptoms [82, 86],self-guided web-based programs that decrease gambling frequency and financial harms; and brief digital motivational interviewing adjuncts to online self-help, which enhance engagement and treatment outcomes [17, 108]. Clinicians should remain attentive to comorbid conditions, particularly substance use, even when clear mental health associations are absent. The observed influence of ACEs on the associations between OGD and substance use highlights the value of screening for childhood adversity in gambling treatment settings. Tailored, trauma-informed approaches may be especially beneficial. These could involve supplementing standard gambling treatments with trauma-focused cognitive behavioral therapy (TF-CBT) modules, shown to reduce both PTSD symptoms and gambling severity more effectively than gambling-only interventions [89],incorporating targeted emotion-regulation training to address rumination as a mediator of gambling severity in individuals with traumatic life histories [42],or adapting principles from trauma-informed group therapies to gambling contexts, thereby strengthening coping skills and reducing substance use and relapse risk. For policymakers, regulatory measures should be responsive to demographic differences in gambling patterns. Responsible gambling initiatives could target specific risk factors, such as substance use vulnerability in men and financial distress in both men and women. Overall, a multidisciplinary approach that integrates clinical psychology, addiction research, and public health strategies is essential for identifying and mitigating the risks associated with online gambling disorder.

### Limitations

Despite its contributions, this study has several important limitations. First, the cross-sectional design precludes any inference of causality between the examined variables. Longitudinal or experimental research is needed to clarify whether the observed associations reflect causal mechanisms. Second, the use of self-reported data for all constructs introduces potential measurement bias. Participants may have underreported or overreported their gambling behaviors and mental health symptoms due to recall inaccuracies or social desirability effects, particularly given the sensitive nature of online gambling and psychological distress. Third, the study relied on total scores for most psychological constructs, except in the case of online gambling disorder, where individual items were modeled within the network analysis. This inconsistency in the level of analysis may have influenced the comparability of centrality and edge-weight estimates across constructs. Relatedly, interpretation of these network parameters should be approached with caution, as the psychometric properties of summed scores differ from those of item-level data. Fourth, the sample consisted entirely of Czech adults recruited through convenience and snowball sampling methods, leading to potential demographic skew and limiting the representativeness of the findings. The specific regulatory, cultural, and societal context of the Czech Republic may not generalize to other countries with different normative frameworks. Finally, the absence of clinical diagnostic data constrains insight into the severity and functional impairment associated with gambling-related issues. Future research should incorporate structured diagnostic interviews and more representative sampling strategies to enhance the validity, generalizability, and clinical relevance of findings.

## Conclusion

This study aimed to examine the relational dynamics between online gambling disorder, psychological distress, suicidal ideation, and substance abuse, while also assessing the impact of adverse childhood experiences (ACEs) and demographic characteristics. By applying network analysis, the research adopted a structural perspective that enabled a nuanced examination of how these psychological and behavioral clusters interact within a broader system of co-occurring risks. The inclusion of ACEs and demographic stratification extended the scope of the analysis by highlighting how early-life adversity and contextual factors shape these associations. The findings emphasize that gambling-related behaviors should not be examined in isolation but considered in relation to other risk-taking behaviors. This integrated approach contributes to the literature by underscoring the importance of contextualizing behavioral addictions within broader psychosocial frameworks. Clinically, therapists can focus on key online gambling symptoms and co-occurring substance use in clients with histories of childhood adversity, using targeted cognitive-behavioral techniques rather than treating gambling in isolation. From a public health perspective, officials can screen for early adversity to identify high-risk gambling and substance use clusters and implement brief community or online interventions addressing how childhood stress fuels these links. For platform designers, monitoring rapid bet increases and embedding reminders of time or spending limits, along with links to self-help resources, may provide timely harm-reduction strategies. Finally, these findings underscore the need for further research into the mechanisms by which OGD manifests differently across sociodemographic groups, advancing more tailored prevention and intervention strategies.

## Supplementary Information

Supplementary Material 1.

Supplementary Material 2.

Supplementary Material 3.

Supplementary Material 4.

Supplementary Material 5.

Supplementary Material 6.

Supplementary Material 7.

Supplementary Material 8.

Supplementary Material 9.

Supplementary Material 10.

Supplementary Material 11.

Supplementary Material 12.

Supplementary Material 13.

Supplementary Material 14.

Supplementary Material 15.

Supplementary Material 16.

Supplementary Material 17.

Supplementary Material 18.

Supplementary Material 19.

Supplementary Material 20.

Supplementary Material 21.

Supplementary Material 22.

Supplementary Material 23.

Supplementary Material 24.

Supplementary Material 25.

Supplementary Material 26.

Supplementary Material 27.

Supplementary Material 28.

Supplementary Material 29.

Supplementary Material 30.

Supplementary Material 31.

Supplementary Material 32.

Supplementary Material 33.

Supplementary Material 34.

Supplementary Material 35.

Supplementary Material 36.

Supplementary Material 37.

Supplementary Material 38.

Supplementary Material 39.

Supplementary Material 40.

Supplementary Material 41.

Supplementary Material 42.

Supplementary Material 43.

Supplementary Material 44.

Supplementary Material 45.

Supplementary Material 46.

Supplementary Material 47.

## Data Availability

Data, programming scripts, and additional resources linked to this research can be accessed through the Zonedo repository at: https://doi.org/10.5281/zenodo.15663319.
