# Supplementary material for: Gambling, trauma, and the mind: a network analysis of online gambling and personal well-being
Source: BMC Psychol. 2025 Nov 5;13:1226. doi: 10.1186/s40359-025-03516-z (PMC12587689; doi:10.1186/s40359-025-03516-z)
Supplement: Supplementary file 1 — Supplementary Material 1. [file 40359_2025_3516_MOESM1_ESM.docx]

# Ordered edge-weight differences of ACE's impact between male and female

| **Var1** | **Var2** | | **Freq** | | **abs_diff** | |
| --- | --- | --- | --- | --- | --- | --- |
| OGD_Q_2 | OGD_Q_6 | -0.1987821990 | | 0.1987821990 | |  |
| OGD_Q_2 | OGD_Q_3 | 0.1464048842 | | 0.1464048842 | |  |
| OGD_Q_5 | OGD_Q_8 | 0.1407071630 | | 0.1407071630 | |  |
| OGD_Q_8 | OGD_Q_10 | -0.1392887297 | | 0.1392887297 | |  |
| OGD_Q_2 | OGD_Q_11 | 0.1234483407 | | 0.1234483407 | |  |
| OGD_Q_10 | OGD_Q_11 | -0.1198312112 | | 0.1198312112 | |  |
| drinking_alcohol | Age | 0.1143434421 | | 0.1143434421 | |  |
| OGD_Q_3 | OGD_Q_8 | 0.1143100154 | | 0.1143100154 | |  |
| stress | Age | 0.1130829610 | | 0.1130829610 | |  |
| OGD_Q_3 | OGD_Q_7 | 0.1070604863 | | 0.1070604863 | |  |
| OGD_Q_3 | OGD_Q_4 | -0.1059153958 | | 0.1059153958 | |  |
| OGD_Q_3 | OGD_Q_9 | -0.1050561636 | | 0.1050561636 | |  |
| OGD_Q_3 | OGD_Q_5 | -0.1046726003 | | 0.1046726003 | |  |
| OGD_Q_6 | OGD_Q_11 | -0.1044099248 | | 0.1044099248 | |  |
| OGD_Q_4 | OGD_Q_10 | 0.0928801588 | | 0.0928801588 | |  |
| OGD_Q_5 | OGD_Q_9 | -0.0896742173 | | 0.0896742173 | |  |
| depression | anxiety | -0.0888792411 | | 0.0888792411 | |  |
| OGD_Q_6 | OGD_Q_8 | -0.0843646149 | | 0.0843646149 | |  |
| OGD_Q_7 | OGD_Q_10 | -0.0829952764 | | 0.0829952764 | |  |
| cannabis_drugs | non_cannabis_drugs | -0.0817686239 | | 0.0817686239 | |  |
| depression | stress | 0.0807574918 | | 0.0807574918 | |  |
| non_cannabis_drugs | prescription_drugs | 0.0802664311 | | 0.0802664311 | |  |
| depression | OGD_Q_1 | -0.0792673360 | | 0.0792673360 | |  |
| OGD_Q_1 | OGD_Q_5 | 0.0788536164 | | 0.0788536164 | |  |
| Education | Income | -0.0784987213 | | 0.0784987213 | |  |
| ACEs | smoking_tobacco | -0.0766689703 | | 0.0766689703 | |  |
| OGD_Q_6 | OGD_Q_7 | 0.0728189417 | | 0.0728189417 | |  |
| OGD_Q_1 | cannabis_drugs | 0.0712568144 | | 0.0712568144 | |  |
| OGD_Q_1 | OGD_Q_8 | 0.0711120545 | | 0.0711120545 | |  |
| OGD_Q_2 | OGD_Q_7 | 0.0684783504 | | 0.0684783504 | |  |
| non_cannabis_drugs | performance_drugs | 0.0653861117 | | 0.0653861117 | |  |
| OGD_Q_5 | performance_drugs | 0.0617875950 | | 0.0617875950 | |  |
| ACEs | Income | 0.0604098972 | | 0.0604098972 | |  |
| OGD_Q_8 | OGD_Q_11 | 0.0596241890 | | 0.0596241890 | |  |
| OGD_Q_2 | OGD_Q_10 | 0.0587771155 | | 0.0587771155 | |  |
| OGD_Q_1 | OGD_Q_3 | 0.0568301158 | | 0.0568301158 | |  |
| smoking_tobacco | Age | 0.0539023324 | | 0.0539023324 | |  |
| cannabis_drugs | prescription_drugs | 0.0538881735 | | 0.0538881735 | |  |
| OGD_Q_3 | OGD_Q_10 | -0.0529177325 | | 0.0529177325 | |  |
| OGD_Q_5 | OGD_Q_7 | 0.0518984534 | | 0.0518984534 | |  |
| stress | suicidal_ideation | -0.0512688354 | | 0.0512688354 | |  |
| Age | Education | -0.0510188760 | | 0.0510188760 | |  |
| OGD_Q_8 | OGD_Q_9 | -0.0507986817 | | 0.0507986817 | |  |
| smoking_tobacco | Education | -0.0504458313 | | 0.0504458313 | |  |
| Age | Income | 0.0503463698 | | 0.0503463698 | |  |
| OGD_Q_2 | OGD_Q_9 | -0.0502481520 | | 0.0502481520 | |  |
| OGD_Q_8 | cannabis_drugs | -0.0472673855 | | 0.0472673855 | |  |
| smoking_tobacco | performance_drugs | -0.0448535347 | | 0.0448535347 | |  |
| OGD_Q_1 | smoking_tobacco | -0.0426241107 | | 0.0426241107 | |  |
| OGD_Q_7 | non_cannabis_drugs | -0.0420777385 | | 0.0420777385 | |  |
| OGD_Q_9 | non_cannabis_drugs | 0.0420634991 | | 0.0420634991 | |  |
| ACEs | stress | -0.0393950928 | | 0.0393950928 | |  |
| stress | OGD_Q_1 | -0.0389320693 | | 0.0389320693 | |  |
| anxiety | prescription_drugs | -0.0383728086 | | 0.0383728086 | |  |
| OGD_Q_9 | OGD_Q_10 | 0.0383222319 | | 0.0383222319 | |  |
| stress | Income | -0.0374844588 | | 0.0374844588 | |  |
| OGD_Q_9 | OGD_Q_11 | 0.0369504307 | | 0.0369504307 | |  |
| smoking_tobacco | non_cannabis_drugs | 0.0367818656 | | 0.0367818656 | |  |
| OGD_Q_8 | Age | -0.0361219521 | | 0.0361219521 | |  |
| suicidal_ideation | Age | -0.0360617050 | | 0.0360617050 | |  |
| OGD_Q_1 | prescription_drugs | -0.0358666337 | | 0.0358666337 | |  |
| ACEs | anxiety | -0.0354421868 | | 0.0354421868 | |  |
| OGD_Q_1 | OGD_Q_11 | -0.0353881798 | | 0.0353881798 | |  |
| anxiety | suicidal_ideation | 0.0352426103 | | 0.0352426103 | |  |
| OGD_Q_2 | OGD_Q_4 | -0.0346760849 | | 0.0346760849 | |  |
| anxiety | OGD_Q_4 | 0.0342879260 | | 0.0342879260 | |  |
| OGD_Q_6 | non_cannabis_drugs | -0.0338870764 | | 0.0338870764 | |  |
| OGD_Q_4 | OGD_Q_5 | -0.0332392504 | | 0.0332392504 | |  |
| OGD_Q_1 | OGD_Q_7 | 0.0313883444 | | 0.0313883444 | |  |
| OGD_Q_3 | OGD_Q_11 | -0.0307420641 | | 0.0307420641 | |  |
| OGD_Q_2 | prescription_drugs | 0.0306130129 | | 0.0306130129 | |  |
| OGD_Q_4 | OGD_Q_6 | 0.0302180038 | | 0.0302180038 | |  |
| OGD_Q_10 | non_cannabis_drugs | 0.0279801793 | | 0.0279801793 | |  |
| depression | smoking_tobacco | -0.0278756690 | | 0.0278756690 | |  |
| OGD_Q_5 | drinking_alcohol | 0.0278401143 | | 0.0278401143 | |  |
| stress | smoking_tobacco | -0.0266160168 | | 0.0266160168 | |  |
| OGD_Q_2 | OGD_Q_5 | -0.0262623414 | | 0.0262623414 | |  |
| OGD_Q_4 | OGD_Q_8 | 0.0256656849 | | 0.0256656849 | |  |
| prescription_drugs | performance_drugs | -0.0251414988 | | 0.0251414988 | |  |
| depression | OGD_Q_6 | -0.0243062109 | | 0.0243062109 | |  |
| ACEs | depression | -0.0240216330 | | 0.0240216330 | |  |
| OGD_Q_10 | cannabis_drugs | 0.0234979804 | | 0.0234979804 | |  |
| prescription_drugs | Age | 0.0233013779 | | 0.0233013779 | |  |
| anxiety | stress | -0.0214295123 | | 0.0214295123 | |  |
| anxiety | performance_drugs | 0.0211693320 | | 0.0211693320 | |  |
| OGD_Q_1 | OGD_Q_4 | 0.0211172019 | | 0.0211172019 | |  |
| suicidal_ideation | smoking_tobacco | -0.0209455181 | | 0.0209455181 | |  |
| OGD_Q_5 | OGD_Q_6 | 0.0208535570 | | 0.0208535570 | |  |
| anxiety | Age | 0.0205927173 | | 0.0205927173 | |  |
| stress | OGD_Q_2 | 0.0201319726 | | 0.0201319726 | |  |
| OGD_Q_10 | performance_drugs | 0.0200099265 | | 0.0200099265 | |  |
| smoking_tobacco | drinking_alcohol | -0.0197942419 | | 0.0197942419 | |  |
| depression | cannabis_drugs | -0.0186477963 | | 0.0186477963 | |  |
| OGD_Q_3 | prescription_drugs | -0.0185794649 | | 0.0185794649 | |  |
| OGD_Q_1 | performance_drugs | -0.0180006844 | | 0.0180006844 | |  |
| OGD_Q_7 | performance_drugs | -0.0173890472 | | 0.0173890472 | |  |
| OGD_Q_4 | drinking_alcohol | -0.0173258573 | | 0.0173258573 | |  |
| depression | suicidal_ideation | -0.0167109300 | | 0.0167109300 | |  |
| anxiety | cannabis_drugs | 0.0160587307 | | 0.0160587307 | |  |
| depression | OGD_Q_3 | -0.0158466175 | | 0.0158466175 | |  |
| stress | OGD_Q_9 | 0.0152769568 | | 0.0152769568 | |  |
| stress | OGD_Q_7 | 0.0152391035 | | 0.0152391035 | |  |
| OGD_Q_2 | OGD_Q_8 | -0.0151170319 | | 0.0151170319 | |  |
| stress | OGD_Q_11 | -0.0149144190 | | 0.0149144190 | |  |
| depression | OGD_Q_10 | -0.0148337387 | | 0.0148337387 | |  |
| depression | OGD_Q_7 | 0.0147226861 | | 0.0147226861 | |  |
| ACEs | suicidal_ideation | -0.0146154340 | | 0.0146154340 | |  |
| OGD_Q_6 | OGD_Q_10 | 0.0142074481 | | 0.0142074481 | |  |
| stress | performance_drugs | 0.0138822010 | | 0.0138822010 | |  |
| OGD_Q_7 | OGD_Q_8 | -0.0131652409 | | 0.0131652409 | |  |
| OGD_Q_5 | non_cannabis_drugs | -0.0129068544 | | 0.0129068544 | |  |
| OGD_Q_4 | performance_drugs | 0.0127719549 | | 0.0127719549 | |  |
| suicidal_ideation | Income | -0.0125561693 | | 0.0125561693 | |  |
| OGD_Q_2 | non_cannabis_drugs | -0.0120089359 | | 0.0120089359 | |  |
| cannabis_drugs | performance_drugs | -0.0116421275 | | 0.0116421275 | |  |
| ACEs | prescription_drugs | -0.0115561859 | | 0.0115561859 | |  |
| OGD_Q_6 | OGD_Q_9 | 0.0113368910 | | 0.0113368910 | |  |
| anxiety | smoking_tobacco | -0.0112110408 | | 0.0112110408 | |  |
| drinking_alcohol | non_cannabis_drugs | -0.0110127392 | | 0.0110127392 | |  |
| OGD_Q_5 | OGD_Q_11 | 0.0107537870 | | 0.0107537870 | |  |
| OGD_Q_6 | prescription_drugs | -0.0101838340 | | 0.0101838340 | |  |
| ACEs | cannabis_drugs | 0.0101246313 | | 0.0101246313 | |  |
| anxiety | drinking_alcohol | 0.0096881056 | | 0.0096881056 | |  |
| OGD_Q_3 | non_cannabis_drugs | -0.0096648927 | | 0.0096648927 | |  |
| OGD_Q_8 | performance_drugs | 0.0095814556 | | 0.0095814556 | |  |
| OGD_Q_1 | Age | -0.0089640707 | | 0.0089640707 | |  |
| OGD_Q_7 | OGD_Q_11 | -0.0089334544 | | 0.0089334544 | |  |
| performance_drugs | Age | -0.0087764480 | | 0.0087764480 | |  |
| OGD_Q_1 | OGD_Q_2 | 0.0082609201 | | 0.0082609201 | |  |
| OGD_Q_4 | OGD_Q_9 | 0.0082440026 | | 0.0082440026 | |  |
| OGD_Q_1 | OGD_Q_10 | 0.0078998154 | | 0.0078998154 | |  |
| depression | OGD_Q_4 | 0.0078083286 | | 0.0078083286 | |  |
| OGD_Q_2 | cannabis_drugs | -0.0072292030 | | 0.0072292030 | |  |
| OGD_Q_4 | OGD_Q_11 | -0.0071052239 | | 0.0071052239 | |  |
| OGD_Q_7 | Income | -0.0066992737 | | 0.0066992737 | |  |
| ACEs | OGD_Q_7 | -0.0065025883 | | 0.0065025883 | |  |
| depression | prescription_drugs | 0.0063838049 | | 0.0063838049 | |  |
| drinking_alcohol | Income | -0.0062040855 | | 0.0062040855 | |  |
| smoking_tobacco | cannabis_drugs | -0.0060053878 | | 0.0060053878 | |  |
| OGD_Q_6 | drinking_alcohol | -0.0054260807 | | 0.0054260807 | |  |
| drinking_alcohol | cannabis_drugs | 0.0052119671 | | 0.0052119671 | |  |
| depression | OGD_Q_9 | -0.0050619611 | | 0.0050619611 | |  |
| OGD_Q_7 | OGD_Q_9 | -0.0042680924 | | 0.0042680924 | |  |
| OGD_Q_1 | OGD_Q_6 | 0.0035812760 | | 0.0035812760 | |  |
| depression | Income | -0.0030356091 | | 0.0030356091 | |  |
| ACEs | OGD_Q_10 | -0.0030213700 | | 0.0030213700 | |  |
| depression | OGD_Q_2 | -0.0027701222 | | 0.0027701222 | |  |
| OGD_Q_4 | OGD_Q_7 | 0.0025102733 | | 0.0025102733 | |  |
| anxiety | Income | -0.0007206177 | | 0.0007206177 | |  |
| OGD_Q_5 | OGD_Q_10 | -0.0006937258 | | 0.0006937258 | |  |
