# Supplementary figures and images for: Gambling, trauma, and the mind: a network analysis of online gambling and personal well-being
Source: BMC Psychol. 2025 Nov 5;13:1226. doi: 10.1186/s40359-025-03516-z (PMC12587689; doi:10.1186/s40359-025-03516-z)

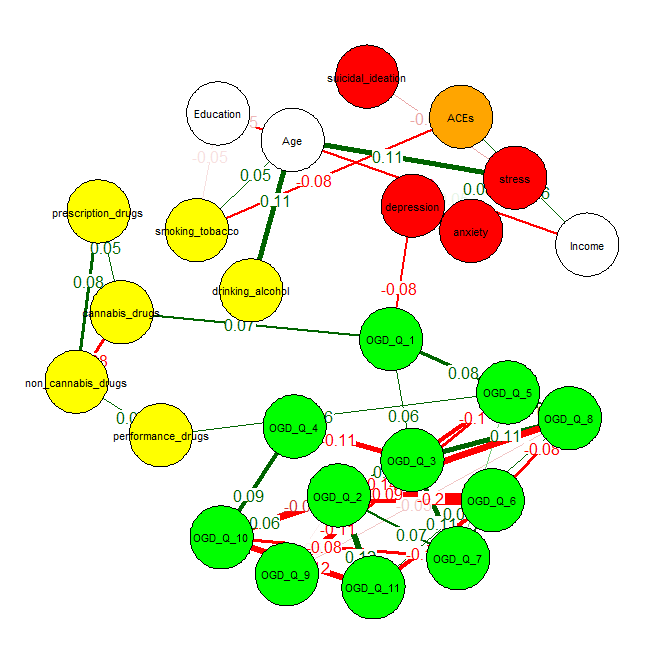

Supplement: Supplementary file 2 — Supplementary Material 2. [file 40359_2025_3516_MOESM2_ESM.png]

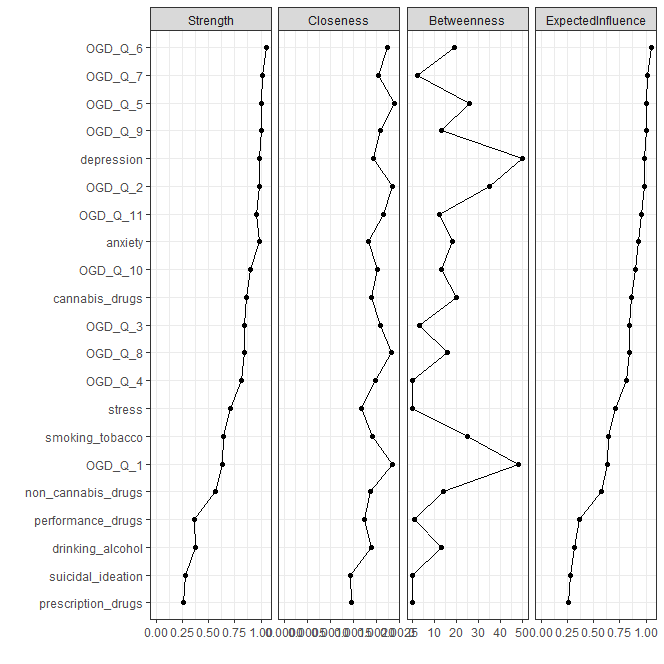

Supplement: Supplementary file 3 — Supplementary Material 3. [file 40359_2025_3516_MOESM3_ESM.png]

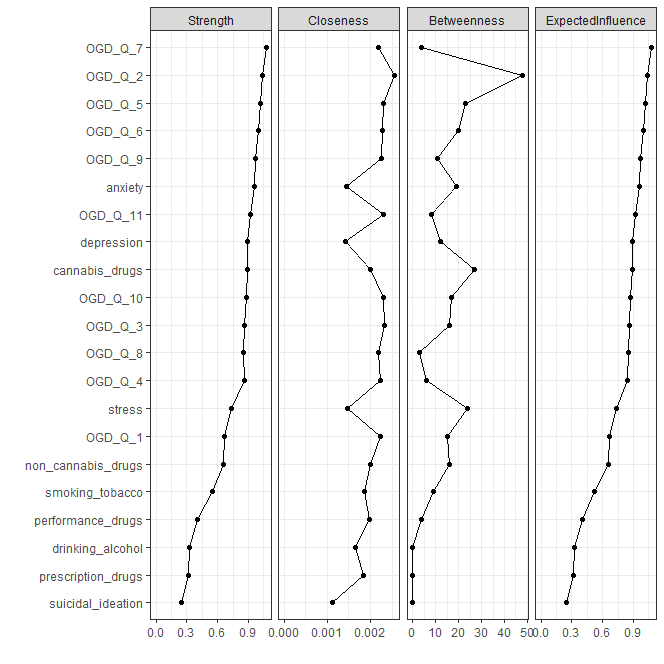

Supplement: Supplementary file 4 — Supplementary Material 4. [file 40359_2025_3516_MOESM4_ESM.png]

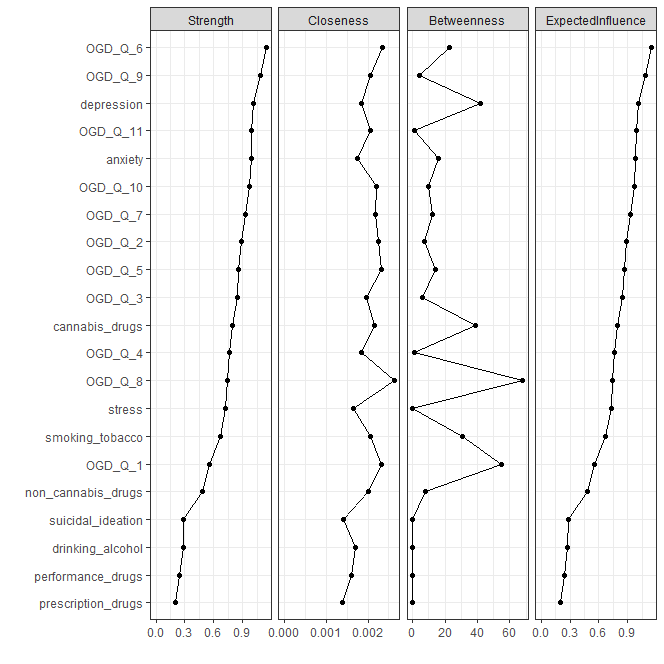

Supplement: Supplementary file 5 — Supplementary Material 5. [file 40359_2025_3516_MOESM5_ESM.png]

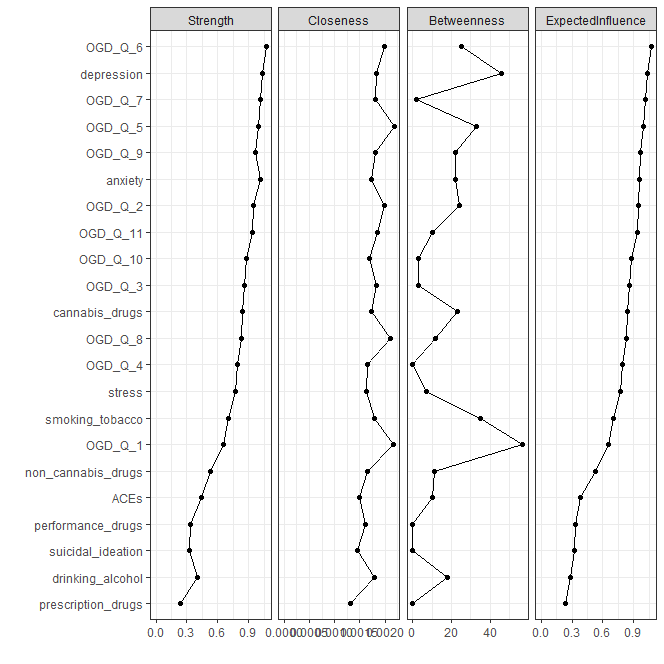

Supplement: Supplementary file 6 — Supplementary Material 6. [file 40359_2025_3516_MOESM6_ESM.png]

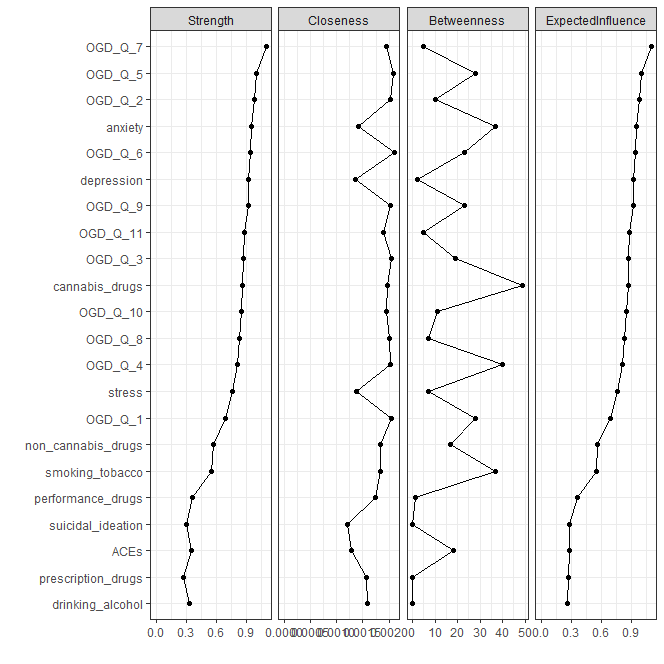

Supplement: Supplementary file 7 — Supplementary Material 7. [file 40359_2025_3516_MOESM7_ESM.png]

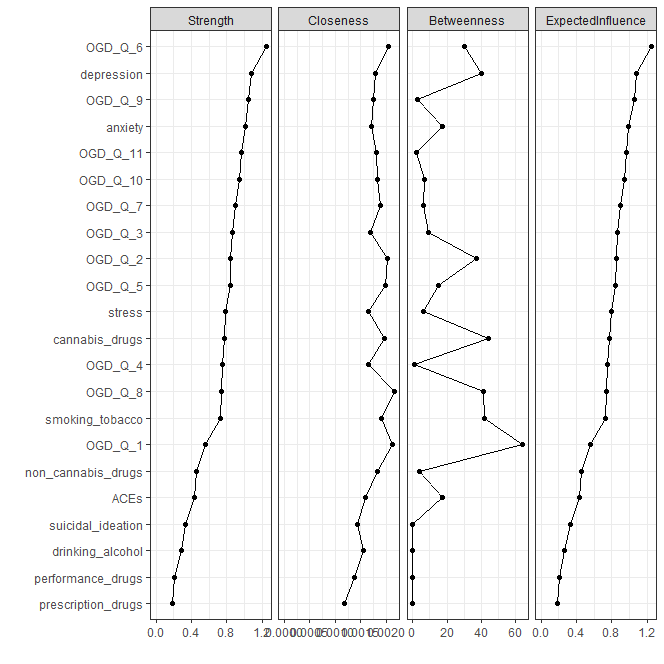

Supplement: Supplementary file 8 — Supplementary Material 8. [file 40359_2025_3516_MOESM8_ESM.png]

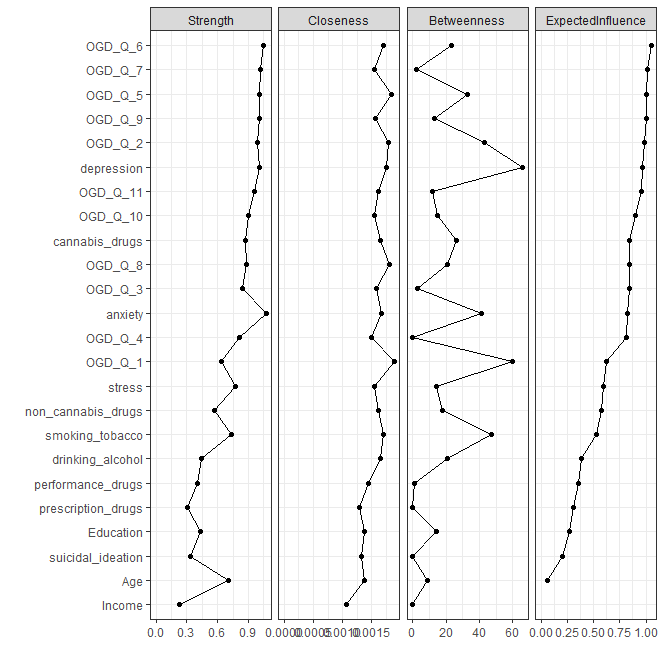

Supplement: Supplementary file 9 — Supplementary Material 9. [file 40359_2025_3516_MOESM9_ESM.png]

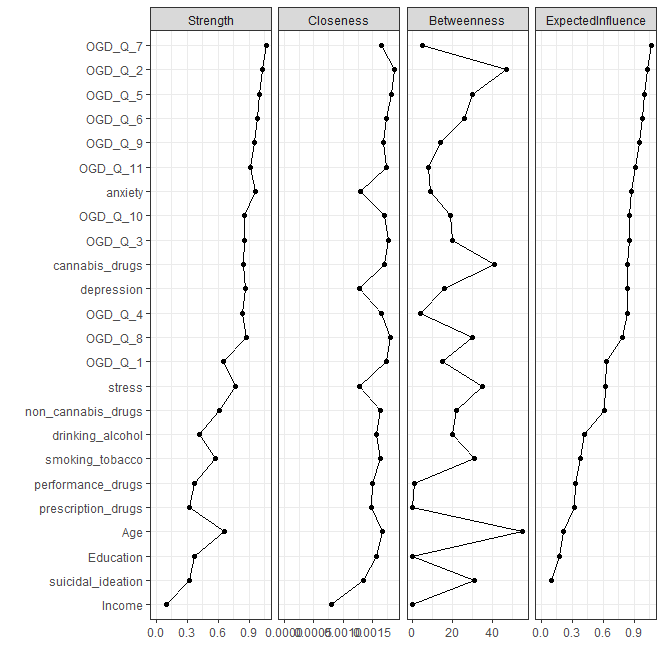

Supplement: Supplementary file 10 — Supplementary Material 10. [file 40359_2025_3516_MOESM10_ESM.png]

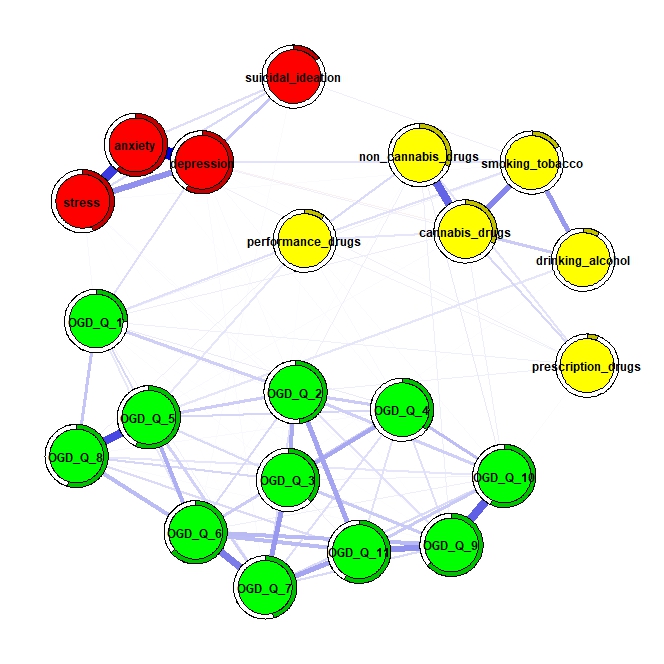

Supplement: Supplementary file 11 — Supplementary Material 11. [file 40359_2025_3516_MOESM11_ESM.jpeg]

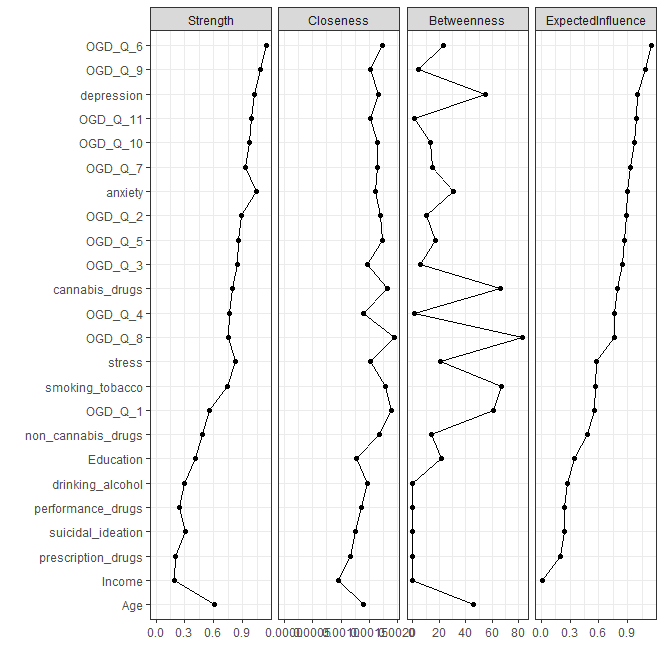

Supplement: Supplementary file 12 — Supplementary Material 12. [file 40359_2025_3516_MOESM12_ESM.png]

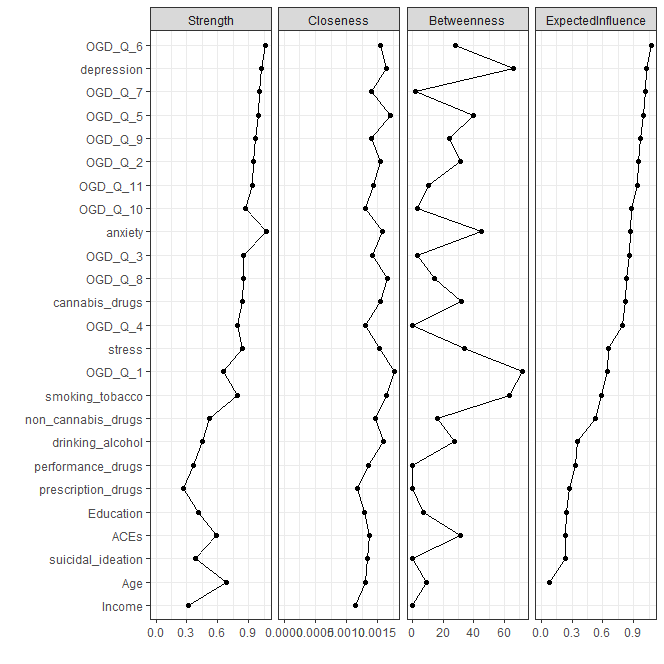

Supplement: Supplementary file 13 — Supplementary Material 13. [file 40359_2025_3516_MOESM13_ESM.png]

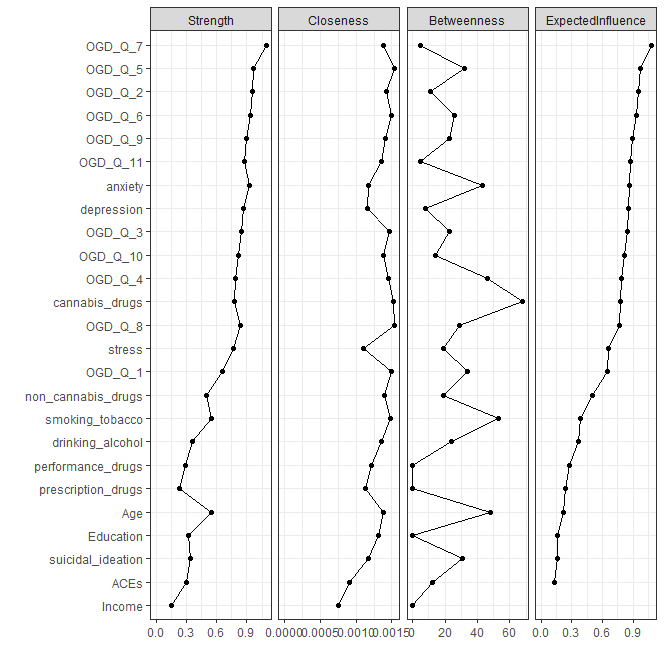

Supplement: Supplementary file 14 — Supplementary Material 14. [file 40359_2025_3516_MOESM14_ESM.png]

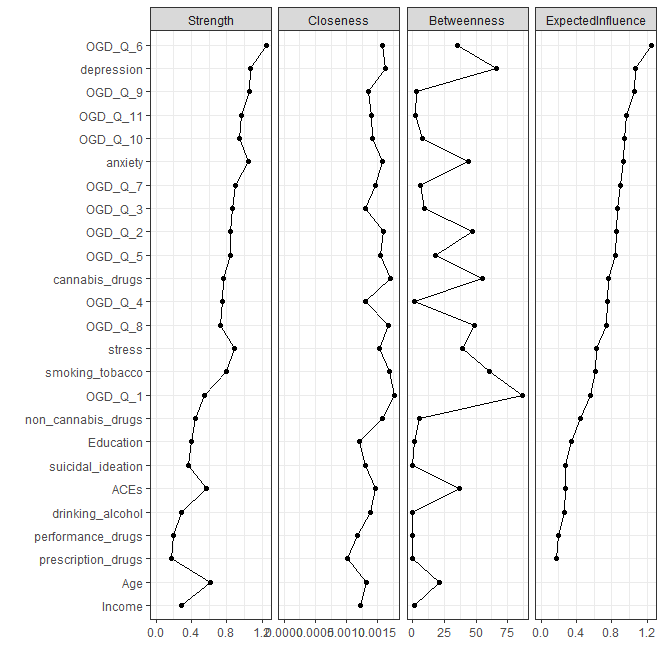

Supplement: Supplementary file 15 — Supplementary Material 15. [file 40359_2025_3516_MOESM15_ESM.png]

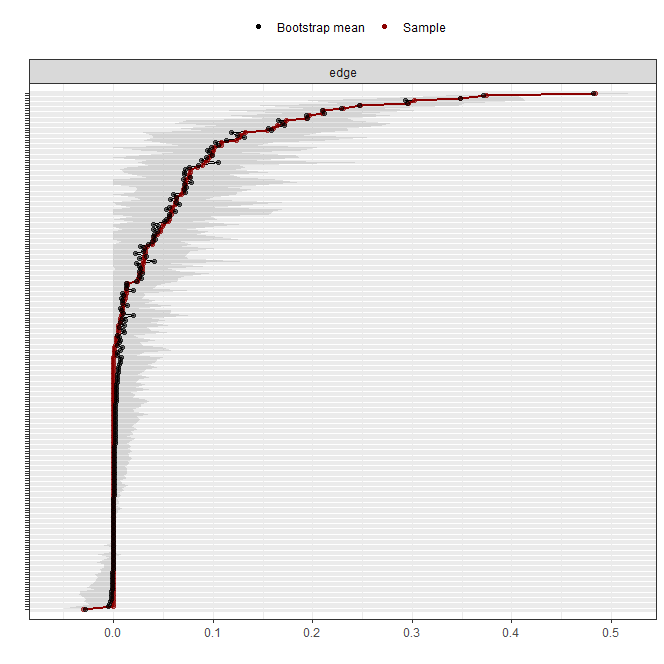

Supplement: Supplementary file 16 — Supplementary Material 16. [file 40359_2025_3516_MOESM16_ESM.png]

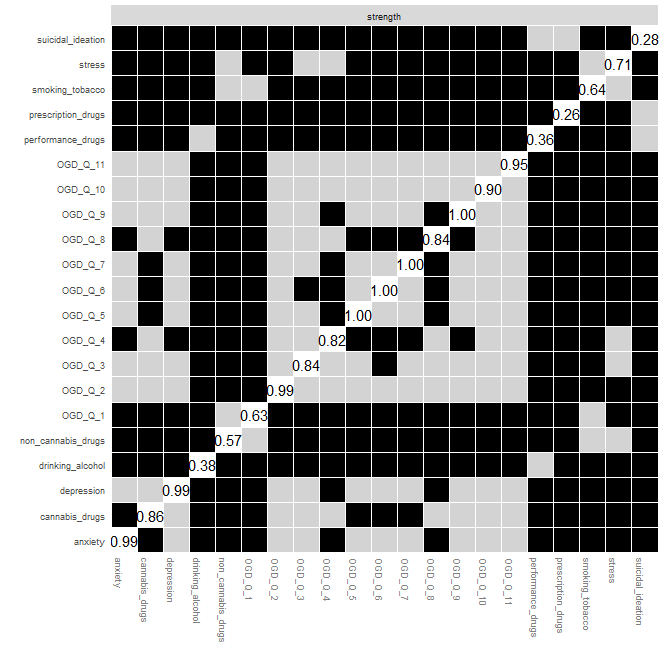

Supplement: Supplementary file 17 — Supplementary Material 17. [file 40359_2025_3516_MOESM17_ESM.png]

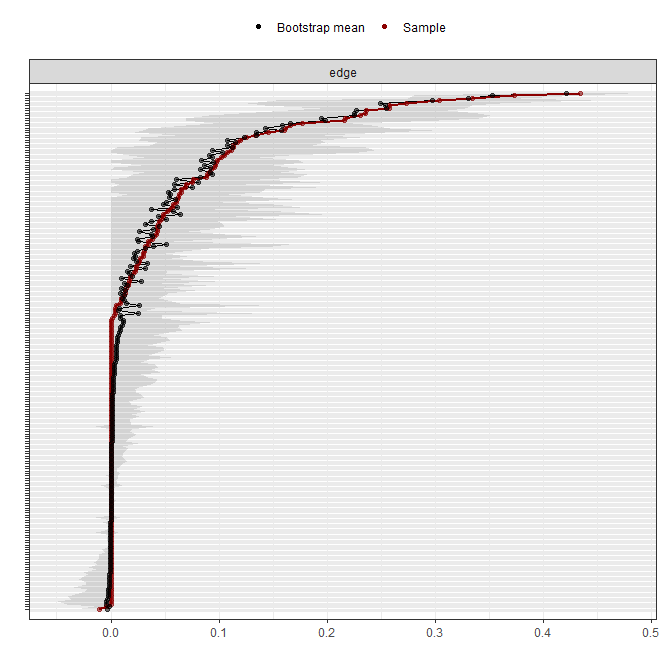

Supplement: Supplementary file 18 — Supplementary Material 18. [file 40359_2025_3516_MOESM18_ESM.png]

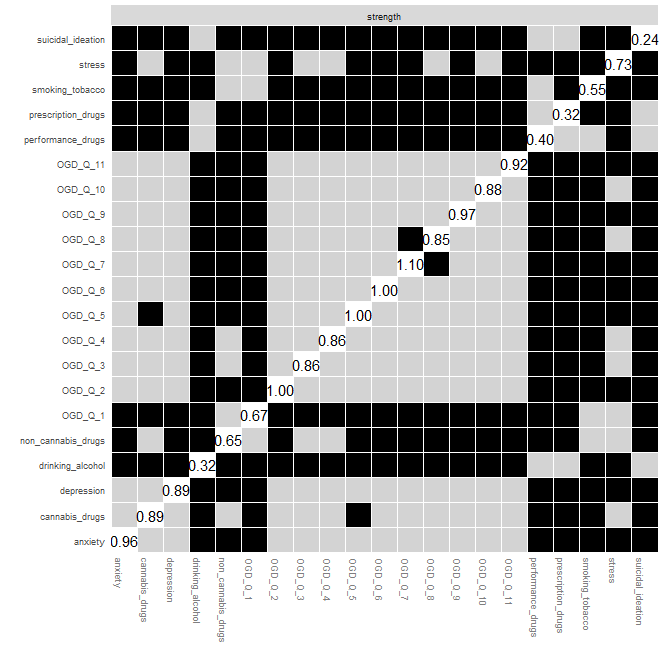

Supplement: Supplementary file 19 — Supplementary Material 19. [file 40359_2025_3516_MOESM19_ESM.png]

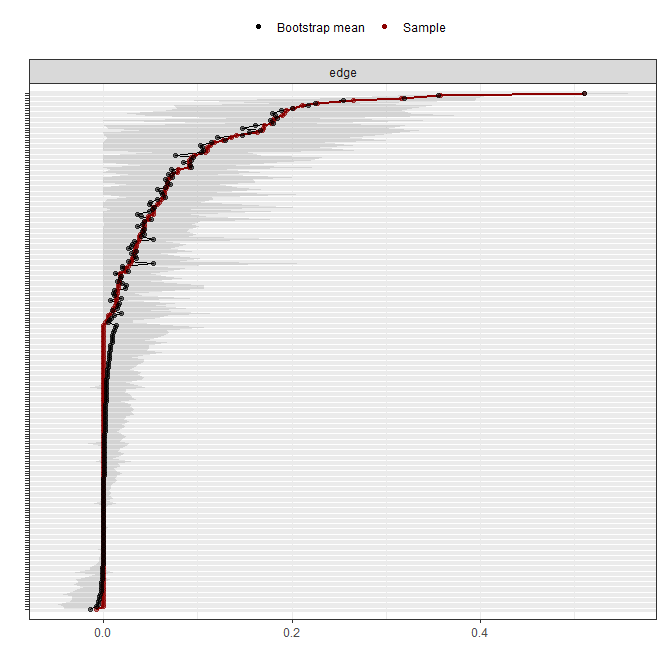

Supplement: Supplementary file 20 — Supplementary Material 20. [file 40359_2025_3516_MOESM20_ESM.png]

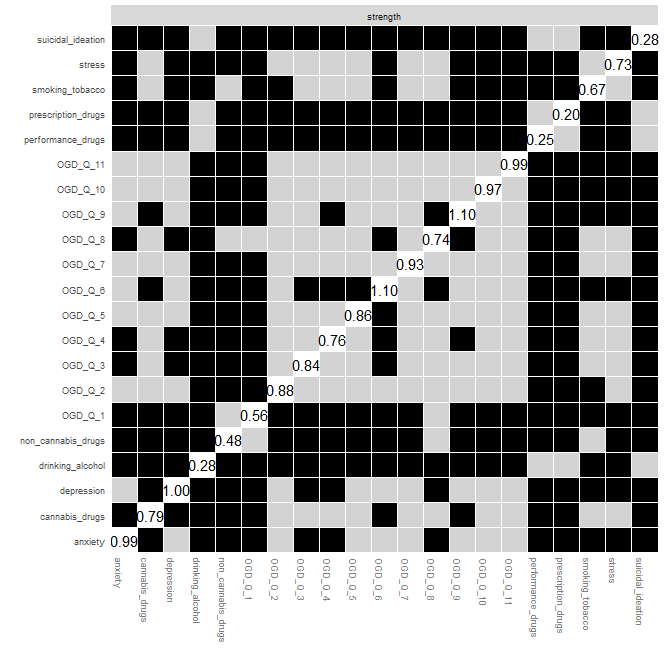

Supplement: Supplementary file 21 — Supplementary Material 21. [file 40359_2025_3516_MOESM21_ESM.png]

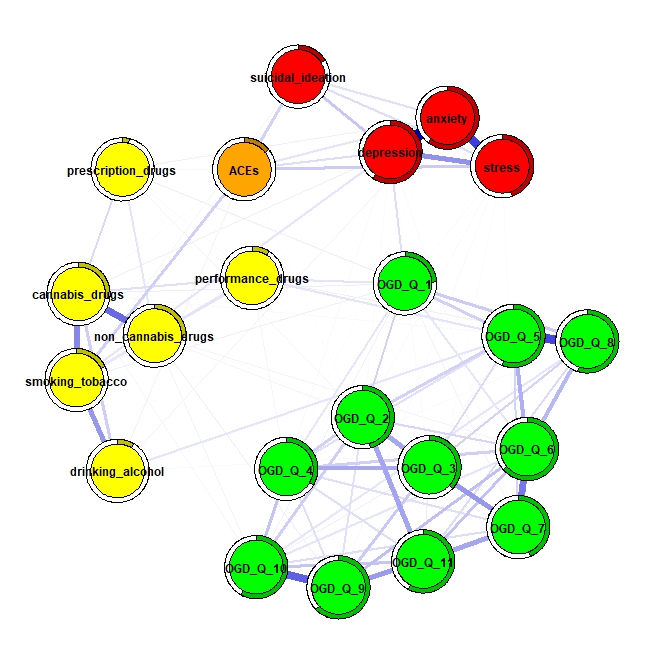

Supplement: Supplementary file 22 — Supplementary Material 22. [file 40359_2025_3516_MOESM22_ESM.jpeg]

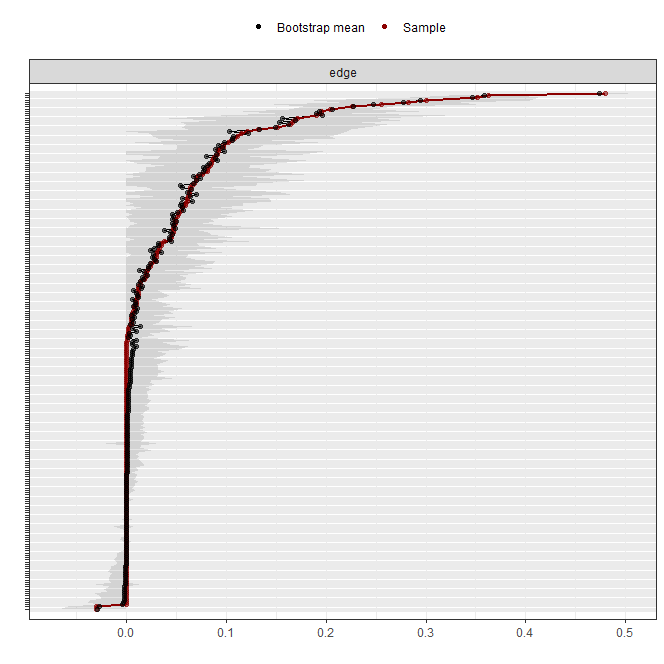

Supplement: Supplementary file 23 — Supplementary Material 23. [file 40359_2025_3516_MOESM23_ESM.png]

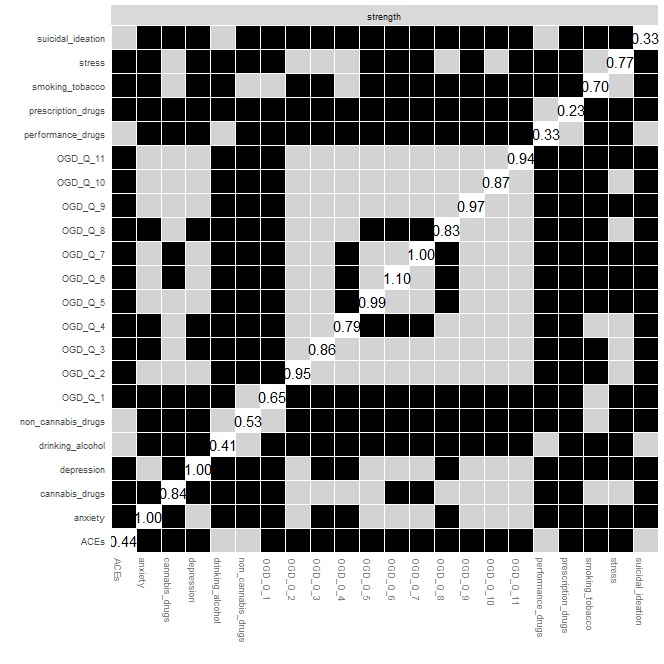

Supplement: Supplementary file 24 — Supplementary Material 24. [file 40359_2025_3516_MOESM24_ESM.png]

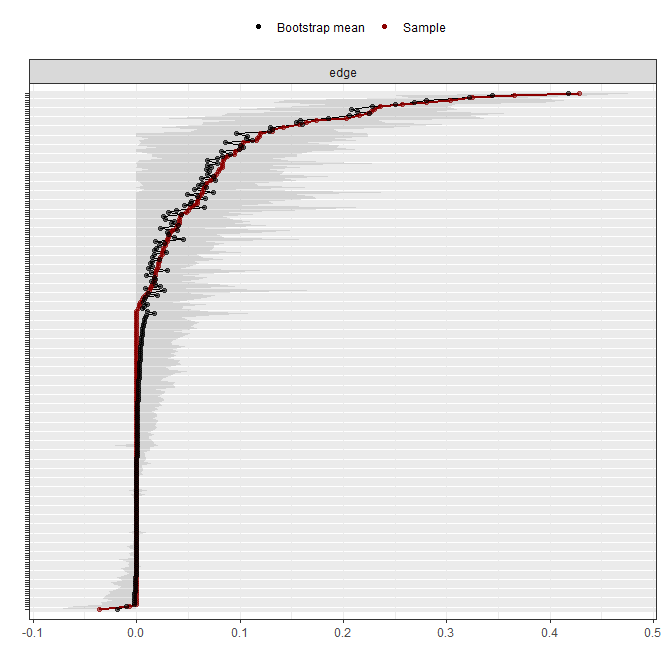

Supplement: Supplementary file 25 — Supplementary Material 25. [file 40359_2025_3516_MOESM25_ESM.png]

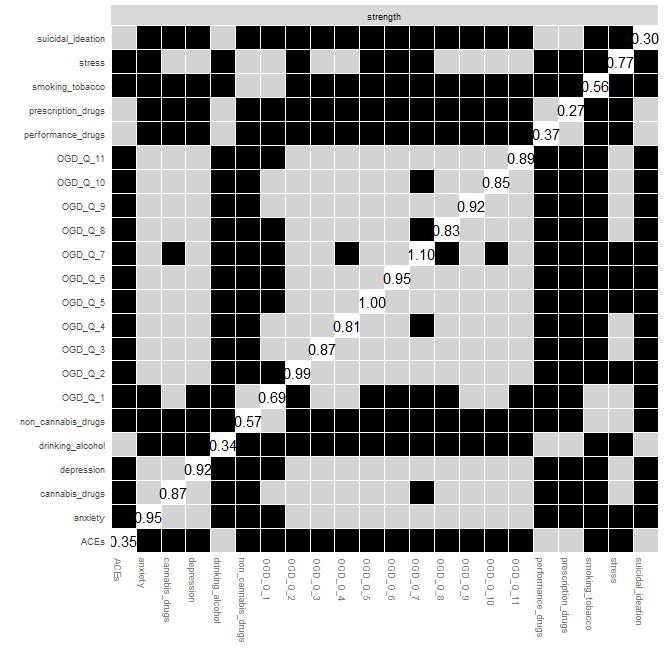

Supplement: Supplementary file 26 — Supplementary Material 26. [file 40359_2025_3516_MOESM26_ESM.png]

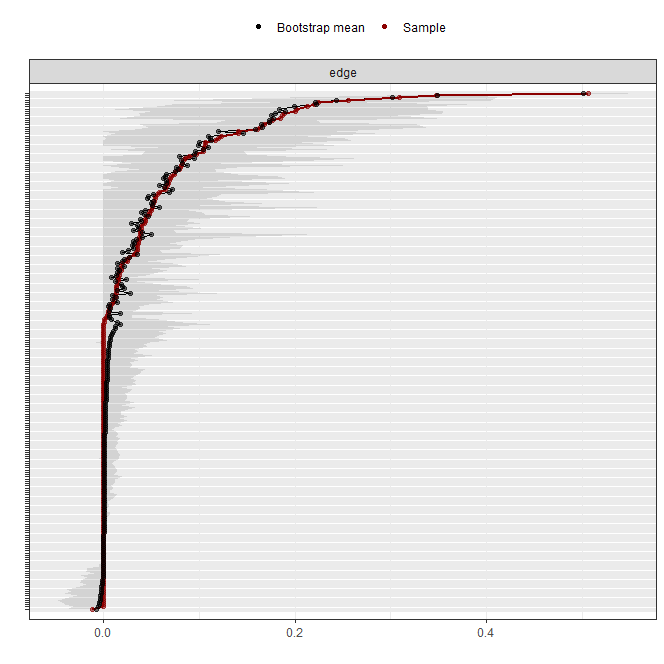

Supplement: Supplementary file 27 — Supplementary Material 27. [file 40359_2025_3516_MOESM27_ESM.png]

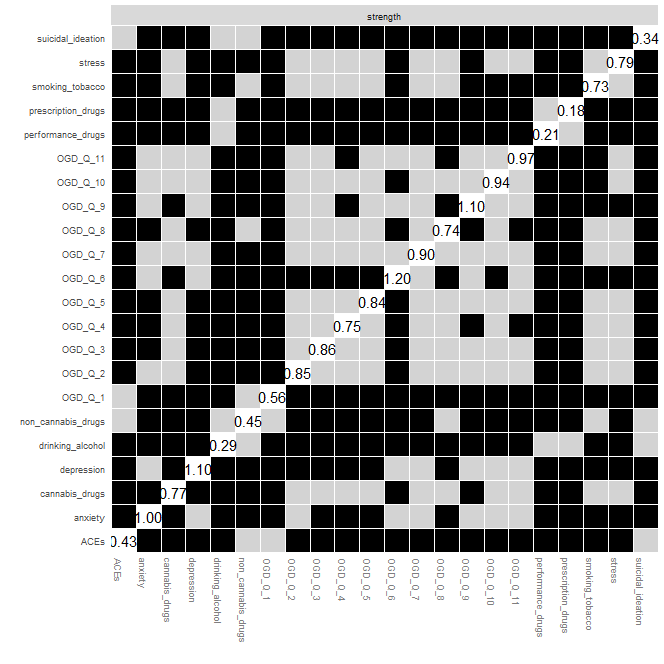

Supplement: Supplementary file 28 — Supplementary Material 28. [file 40359_2025_3516_MOESM28_ESM.png]

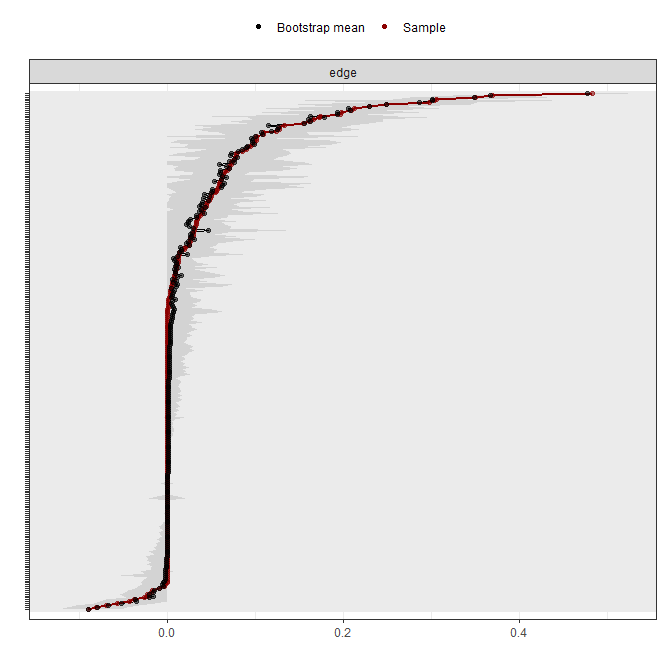

Supplement: Supplementary file 29 — Supplementary Material 29. [file 40359_2025_3516_MOESM29_ESM.png]

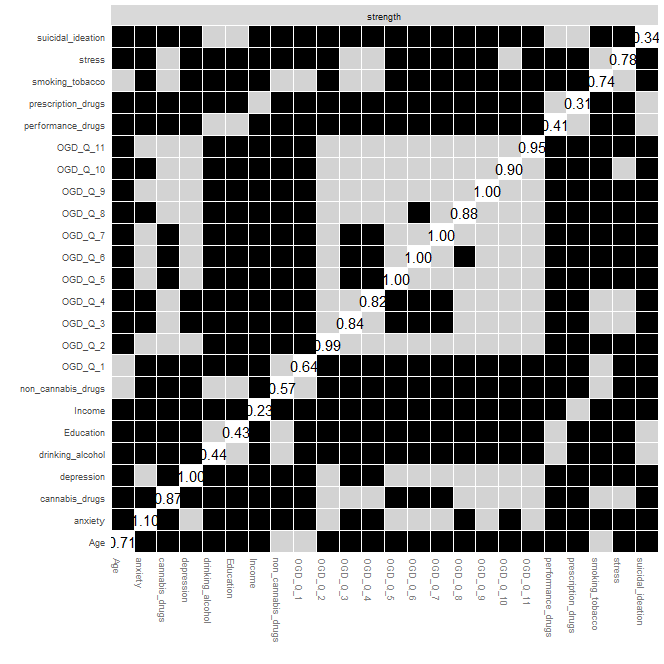

Supplement: Supplementary file 30 — Supplementary Material 30. [file 40359_2025_3516_MOESM30_ESM.png]

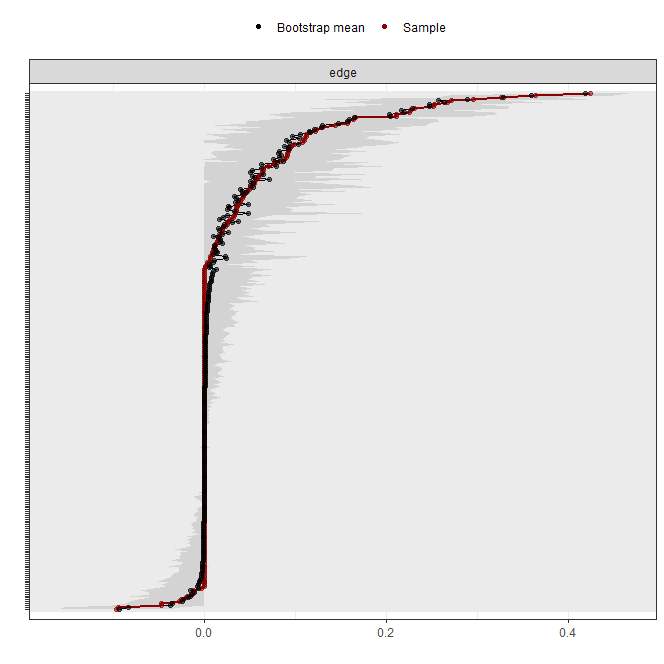

Supplement: Supplementary file 31 — Supplementary Material 31. [file 40359_2025_3516_MOESM31_ESM.png]

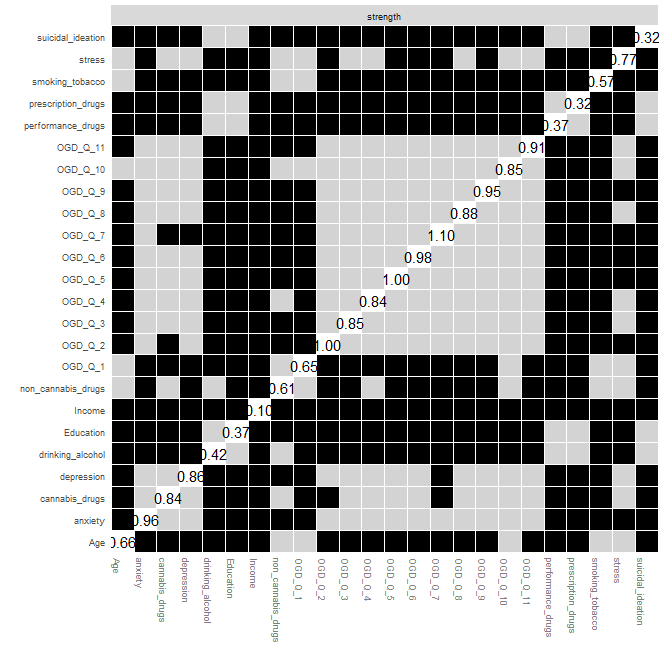

Supplement: Supplementary file 32 — Supplementary Material 32. [file 40359_2025_3516_MOESM32_ESM.png]

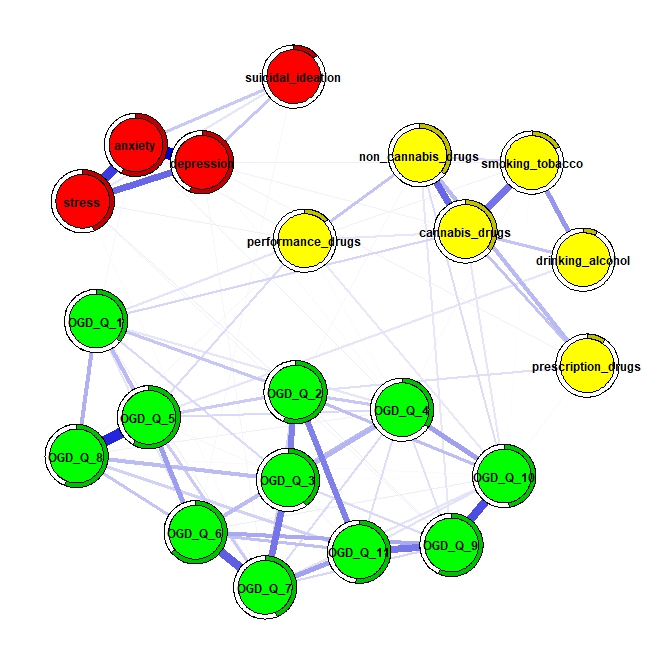

Supplement: Supplementary file 33 — Supplementary Material 33. [file 40359_2025_3516_MOESM33_ESM.jpeg]

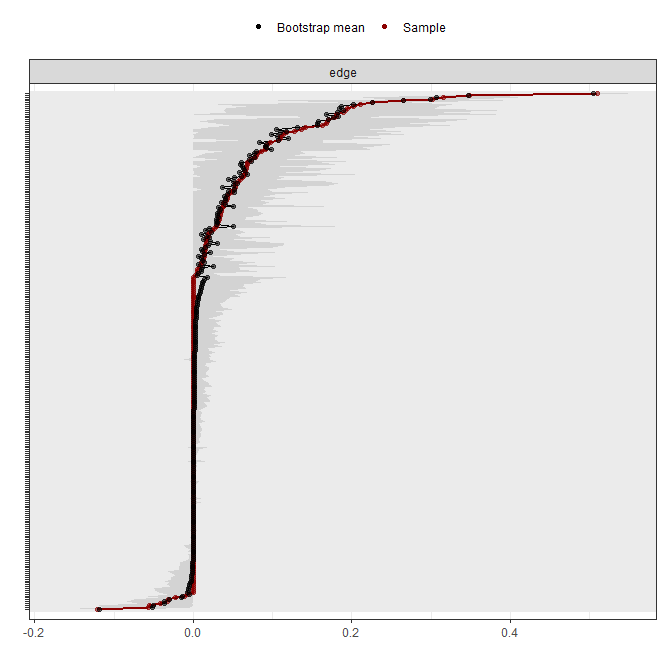

Supplement: Supplementary file 34 — Supplementary Material 34. [file 40359_2025_3516_MOESM34_ESM.png]

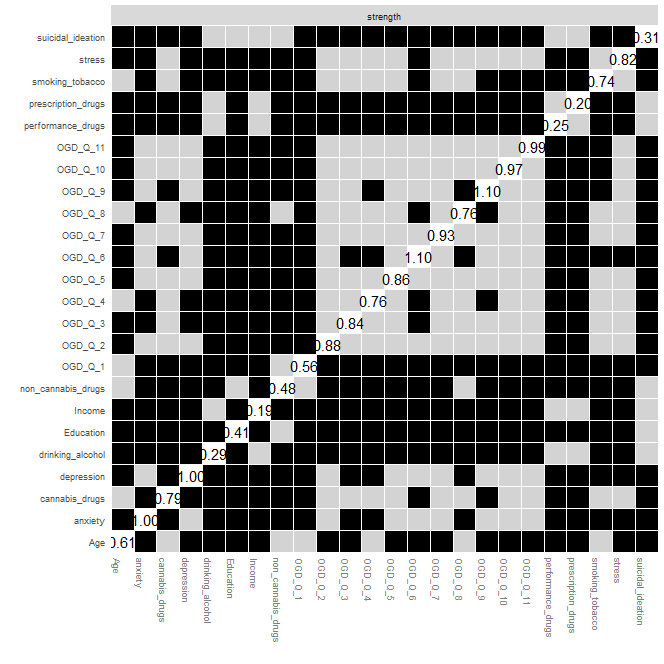

Supplement: Supplementary file 35 — Supplementary Material 35. [file 40359_2025_3516_MOESM35_ESM.png]

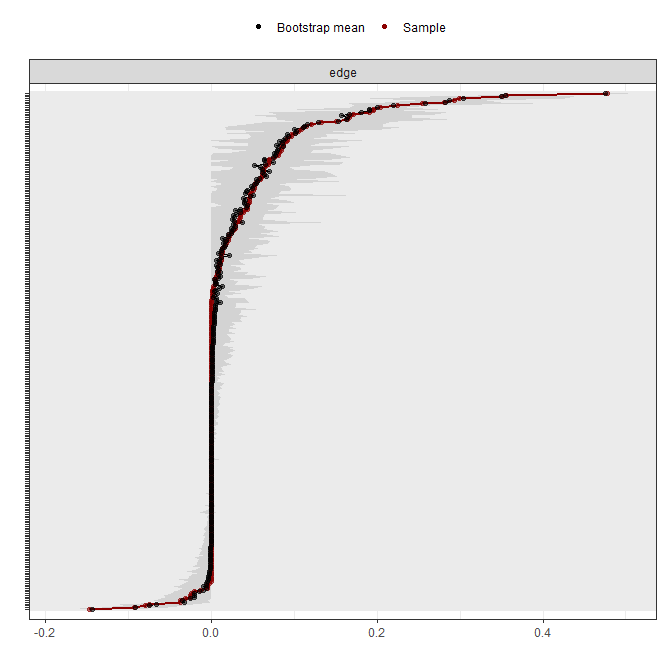

Supplement: Supplementary file 36 — Supplementary Material 36. [file 40359_2025_3516_MOESM36_ESM.png]

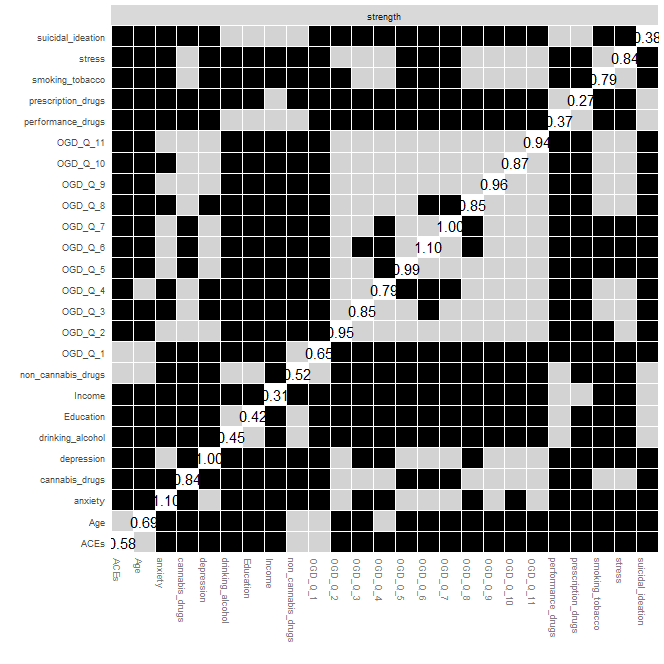

Supplement: Supplementary file 37 — Supplementary Material 37. [file 40359_2025_3516_MOESM37_ESM.png]

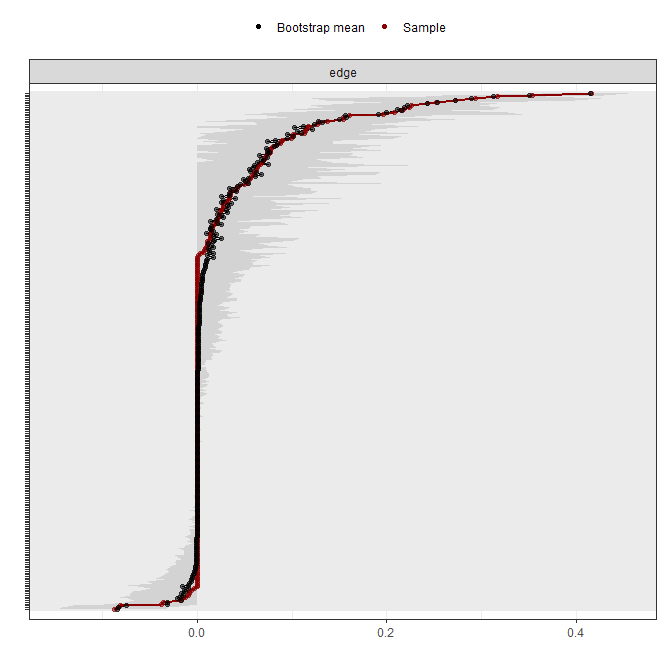

Supplement: Supplementary file 38 — Supplementary Material 38. [file 40359_2025_3516_MOESM38_ESM.png]

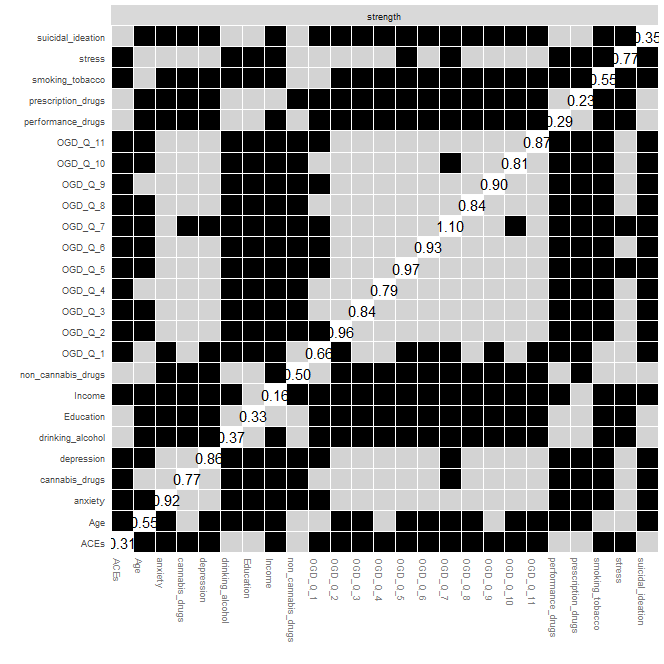

Supplement: Supplementary file 39 — Supplementary Material 39. [file 40359_2025_3516_MOESM39_ESM.png]

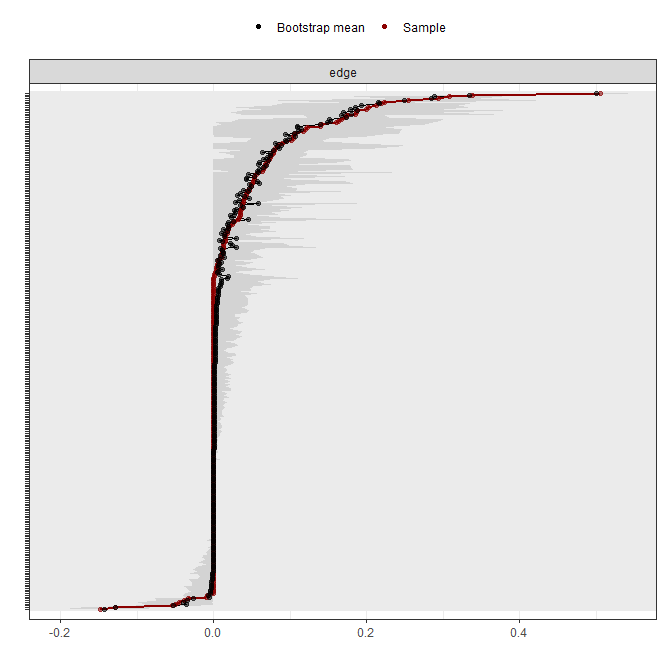

Supplement: Supplementary file 40 — Supplementary Material 40. [file 40359_2025_3516_MOESM40_ESM.png]

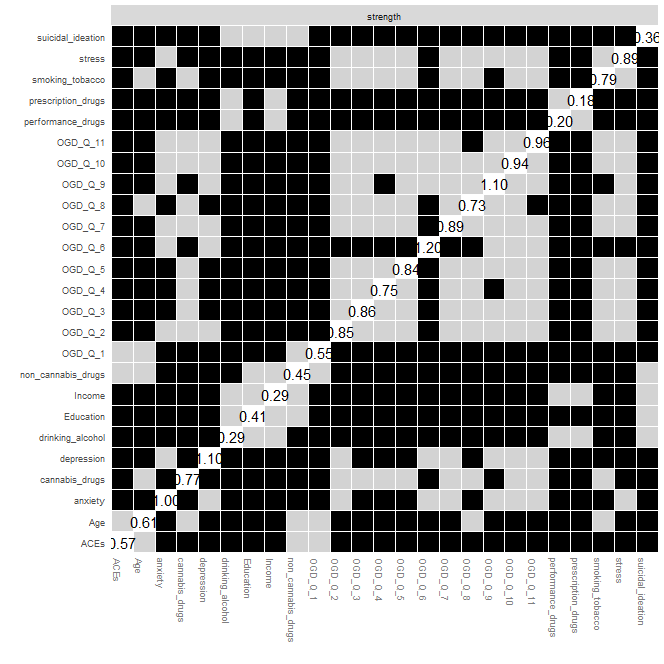

Supplement: Supplementary file 41 — Supplementary Material 41. [file 40359_2025_3516_MOESM41_ESM.png]

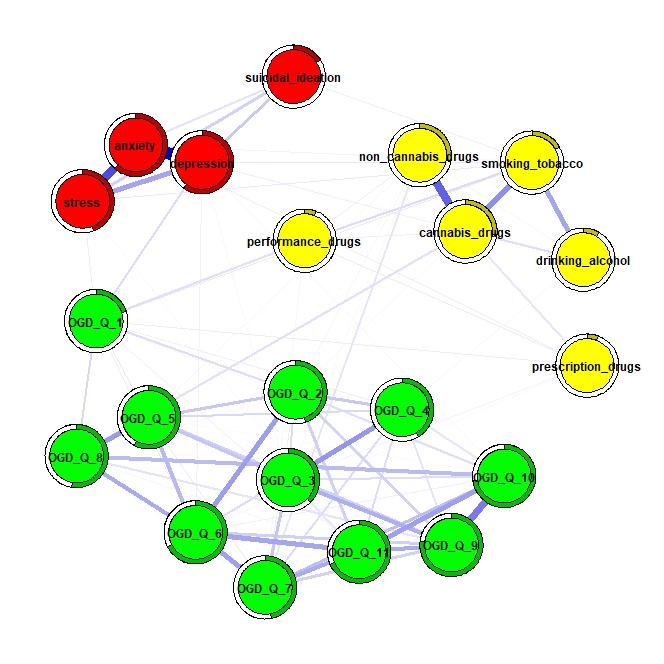

Supplement: Supplementary file 42 — Supplementary Material 42. [file 40359_2025_3516_MOESM42_ESM.jpeg]

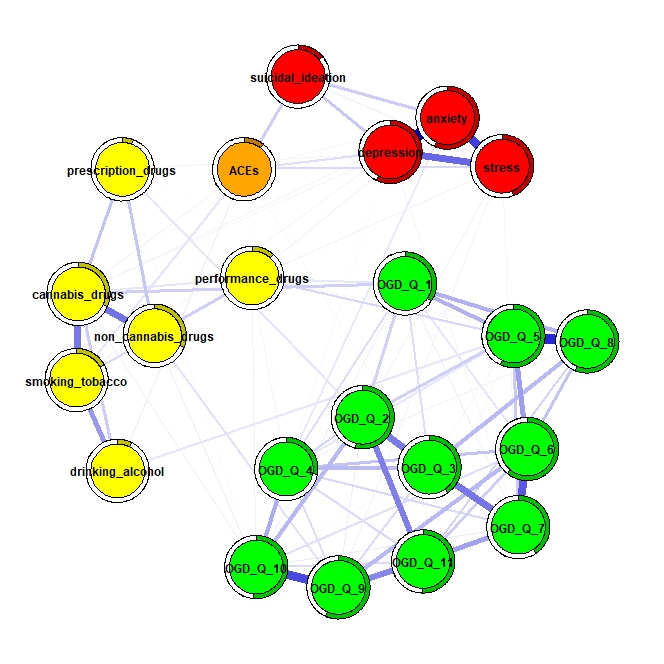

Supplement: Supplementary file 43 — Supplementary Material 43. [file 40359_2025_3516_MOESM43_ESM.jpeg]

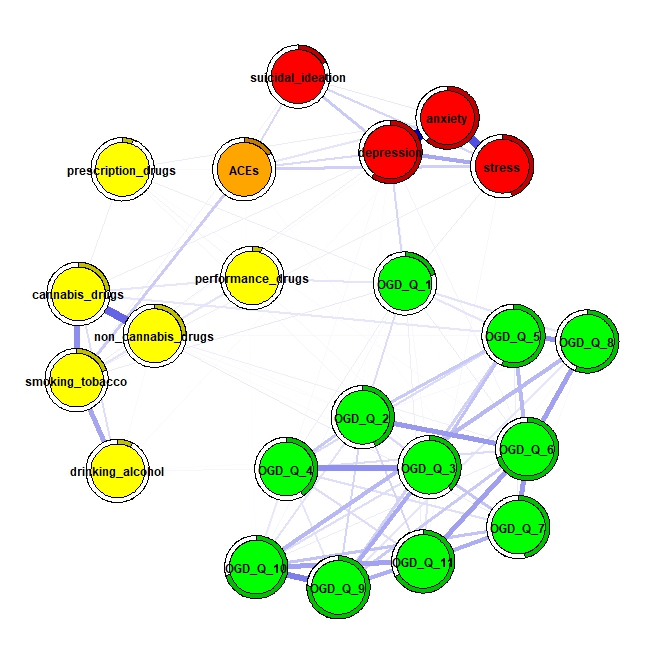

Supplement: Supplementary file 44 — Supplementary Material 44. [file 40359_2025_3516_MOESM44_ESM.jpeg]
