# Supplementary material for: Gambling, trauma, and the mind: a network analysis of online gambling and personal well-being
Source: BMC Psychol. 2025 Nov 5;13:1226. doi: 10.1186/s40359-025-03516-z (PMC12587689; doi:10.1186/s40359-025-03516-z)
Supplement: Supplementary file 45 — Supplementary Material 45. [file 40359_2025_3516_MOESM45_ESM.docx]

# Ordered edge-weight differences (full sample adjusted networks)

| **Var1** | **Var2** | | **Freq** | | **abs_diff** | |
| --- | --- | --- | --- | --- | --- | --- |
| OGD_Q_9 | OGD_Q_11 | -0.0212641920 | | 0.0212641920 | |  |
| anxiety | Income | 0.0194256341 | | 0.0194256341 | |  |
| OGD_Q_4 | OGD_Q_5 | -0.0186041892 | | 0.0186041892 | |  |
| non_cannabis_drugs | performance_drugs | -0.0179595772 | | 0.0179595772 | |  |
| OGD_Q_6 | OGD_Q_10 | 0.0174474192 | | 0.0174474192 | |  |
| OGD_Q_6 | non_cannabis_drugs | 0.0167111285 | | 0.0167111285 | |  |
| OGD_Q_10 | non_cannabis_drugs | -0.0162907535 | | 0.0162907535 | |  |
| cannabis_drugs | non_cannabis_drugs | -0.0155752179 | | 0.0155752179 | |  |
| OGD_Q_1 | OGD_Q_10 | 0.0152300374 | | 0.0152300374 | |  |
| OGD_Q_4 | OGD_Q_10 | -0.0146918026 | | 0.0146918026 | |  |
| suicidal_ideation | smoking_tobacco | -0.0146277038 | | 0.0146277038 | |  |
| depression | Income | 0.0137989215 | | 0.0137989215 | |  |
| OGD_Q_10 | cannabis_drugs | -0.0137520410 | | 0.0137520410 | |  |
| prescription_drugs | performance_drugs | -0.0134937922 | | 0.0134937922 | |  |
| anxiety | stress | -0.0130370409 | | 0.0130370409 | |  |
| OGD_Q_2 | OGD_Q_4 | -0.0125575762 | | 0.0125575762 | |  |
| Age | Income | 0.0120978355 | | 0.0120978355 | |  |
| Age | Education | -0.0120672429 | | 0.0120672429 | |  |
| OGD_Q_1 | OGD_Q_8 | -0.0118410572 | | 0.0118410572 | |  |
| OGD_Q_6 | OGD_Q_9 | -0.0115731686 | | 0.0115731686 | |  |
| OGD_Q_6 | OGD_Q_11 | -0.0114767535 | | 0.0114767535 | |  |
| OGD_Q_1 | OGD_Q_5 | 0.0114614727 | | 0.0114614727 | |  |
| stress | suicidal_ideation | -0.0114083557 | | 0.0114083557 | |  |
| OGD_Q_8 | Income | -0.0108066244 | | 0.0108066244 | |  |
| OGD_Q_2 | prescription_drugs | -0.0107736375 | | 0.0107736375 | |  |
| non_cannabis_drugs | prescription_drugs | -0.0107560958 | | 0.0107560958 | |  |
| OGD_Q_10 | OGD_Q_11 | 0.0107317801 | | 0.0107317801 | |  |
| OGD_Q_2 | non_cannabis_drugs | -0.0107101456 | | 0.0107101456 | |  |
| cannabis_drugs | performance_drugs | 0.0098371230 | | 0.0098371230 | |  |
| OGD_Q_2 | OGD_Q_5 | -0.0091984647 | | 0.0091984647 | |  |
| OGD_Q_10 | performance_drugs | -0.0090597615 | | 0.0090597615 | |  |
| OGD_Q_10 | prescription_drugs | -0.0089862359 | | 0.0089862359 | |  |
| depression | smoking_tobacco | -0.0089580718 | | 0.0089580718 | |  |
| OGD_Q_7 | OGD_Q_10 | -0.0088229106 | | 0.0088229106 | |  |
| OGD_Q_2 | OGD_Q_7 | 0.0083840633 | | 0.0083840633 | |  |
| drinking_alcohol | cannabis_drugs | -0.0081225566 | | 0.0081225566 | |  |
| OGD_Q_1 | OGD_Q_4 | 0.0081083675 | | 0.0081083675 | |  |
| OGD_Q_1 | performance_drugs | -0.0077732038 | | 0.0077732038 | |  |
| anxiety | suicidal_ideation | -0.0077097278 | | 0.0077097278 | |  |
| smoking_tobacco | performance_drugs | -0.0073731828 | | 0.0073731828 | |  |
| OGD_Q_1 | OGD_Q_2 | -0.0072471186 | | 0.0072471186 | |  |
| suicidal_ideation | OGD_Q_6 | -0.0068986513 | | 0.0068986513 | |  |
| cannabis_drugs | prescription_drugs | -0.0064301717 | | 0.0064301717 | |  |
| OGD_Q_6 | OGD_Q_7 | 0.0060932704 | | 0.0060932704 | |  |
| OGD_Q_6 | drinking_alcohol | 0.0057481067 | | 0.0057481067 | |  |
| OGD_Q_6 | OGD_Q_8 | 0.0055791155 | | 0.0055791155 | |  |
| suicidal_ideation | Age | -0.0055531458 | | 0.0055531458 | |  |
| OGD_Q_5 | OGD_Q_9 | 0.0055403176 | | 0.0055403176 | |  |
| Education | Income | -0.0055324264 | | 0.0055324264 | |  |
| smoking_tobacco | cannabis_drugs | -0.0054397032 | | 0.0054397032 | |  |
| OGD_Q_4 | OGD_Q_6 | -0.0052677210 | | 0.0052677210 | |  |
| anxiety | OGD_Q_4 | 0.0052379057 | | 0.0052379057 | |  |
| OGD_Q_3 | OGD_Q_10 | 0.0052035966 | | 0.0052035966 | |  |
| cannabis_drugs | Age | 0.0050609062 | | 0.0050609062 | |  |
| OGD_Q_2 | OGD_Q_3 | 0.0049712891 | | 0.0049712891 | |  |
| depression | stress | -0.0049688651 | | 0.0049688651 | |  |
| OGD_Q_4 | OGD_Q_9 | 0.0048069299 | | 0.0048069299 | |  |
| OGD_Q_2 | cannabis_drugs | 0.0047851035 | | 0.0047851035 | |  |
| OGD_Q_1 | prescription_drugs | 0.0047564080 | | 0.0047564080 | |  |
| depression | prescription_drugs | -0.0046358731 | | 0.0046358731 | |  |
| OGD_Q_8 | OGD_Q_10 | -0.0045506066 | | 0.0045506066 | |  |
| OGD_Q_8 | OGD_Q_9 | -0.0045384647 | | 0.0045384647 | |  |
| OGD_Q_7 | OGD_Q_11 | -0.0043986162 | | 0.0043986162 | |  |
| depression | anxiety | -0.0043779980 | | 0.0043779980 | |  |
| OGD_Q_1 | smoking_tobacco | 0.0043209894 | | 0.0043209894 | |  |
| OGD_Q_8 | Age | 0.0042854675 | | 0.0042854675 | |  |
| OGD_Q_4 | performance_drugs | 0.0042643242 | | 0.0042643242 | |  |
| smoking_tobacco | non_cannabis_drugs | 0.0037607206 | | 0.0037607206 | |  |
| OGD_Q_3 | OGD_Q_8 | 0.0037576829 | | 0.0037576829 | |  |
| OGD_Q_5 | performance_drugs | 0.0037020484 | | 0.0037020484 | |  |
| performance_drugs | Age | 0.0036221499 | | 0.0036221499 | |  |
| OGD_Q_5 | OGD_Q_6 | -0.0034614364 | | 0.0034614364 | |  |
| OGD_Q_4 | OGD_Q_11 | 0.0033471008 | | 0.0033471008 | |  |
| OGD_Q_2 | OGD_Q_6 | 0.0032072598 | | 0.0032072598 | |  |
| OGD_Q_5 | OGD_Q_7 | -0.0031876785 | | 0.0031876785 | |  |
| anxiety | OGD_Q_9 | -0.0031352947 | | 0.0031352947 | |  |
| OGD_Q_9 | OGD_Q_10 | -0.0028857710 | | 0.0028857710 | |  |
| depression | suicidal_ideation | -0.0028616170 | | 0.0028616170 | |  |
| OGD_Q_2 | OGD_Q_9 | -0.0028139433 | | 0.0028139433 | |  |
| stress | Age | -0.0027808131 | | 0.0027808131 | |  |
| OGD_Q_2 | OGD_Q_11 | -0.0027642319 | | 0.0027642319 | |  |
| OGD_Q_1 | OGD_Q_6 | 0.0026059536 | | 0.0026059536 | |  |
| performance_drugs | Income | 0.0025663557 | | 0.0025663557 | |  |
| stress | OGD_Q_2 | -0.0024689398 | | 0.0024689398 | |  |
| OGD_Q_1 | OGD_Q_9 | -0.0024678249 | | 0.0024678249 | |  |
| stress | OGD_Q_7 | 0.0023865926 | | 0.0023865926 | |  |
| stress | smoking_tobacco | -0.0023730673 | | 0.0023730673 | |  |
| cannabis_drugs | Education | -0.0023669830 | | 0.0023669830 | |  |
| drinking_alcohol | Income | -0.0023021359 | | 0.0023021359 | |  |
| anxiety | drinking_alcohol | 0.0022640801 | | 0.0022640801 | |  |
| stress | OGD_Q_11 | 0.0022484720 | | 0.0022484720 | |  |
| stress | OGD_Q_1 | -0.0022099444 | | 0.0022099444 | |  |
| OGD_Q_1 | Age | 0.0019878964 | | 0.0019878964 | |  |
| OGD_Q_5 | OGD_Q_8 | 0.0019151689 | | 0.0019151689 | |  |
| prescription_drugs | Age | -0.0018935174 | | 0.0018935174 | |  |
| OGD_Q_11 | prescription_drugs | 0.0018928011 | | 0.0018928011 | |  |
| depression | OGD_Q_2 | -0.0017710473 | | 0.0017710473 | |  |
| OGD_Q_7 | OGD_Q_9 | -0.0016998803 | | 0.0016998803 | |  |
| OGD_Q_7 | performance_drugs | 0.0016823684 | | 0.0016823684 | |  |
| depression | OGD_Q_1 | 0.0016260484 | | 0.0016260484 | |  |
| OGD_Q_3 | OGD_Q_9 | 0.0015783416 | | 0.0015783416 | |  |
| OGD_Q_8 | OGD_Q_11 | -0.0015702782 | | 0.0015702782 | |  |
| OGD_Q_5 | drinking_alcohol | -0.0014734569 | | 0.0014734569 | |  |
| OGD_Q_3 | OGD_Q_7 | -0.0014527046 | | 0.0014527046 | |  |
| smoking_tobacco | Age | -0.0013194731 | | 0.0013194731 | |  |
| OGD_Q_3 | OGD_Q_5 | -0.0012766889 | | 0.0012766889 | |  |
| OGD_Q_3 | OGD_Q_4 | -0.0012666527 | | 0.0012666527 | |  |
| anxiety | Age | 0.0012351081 | | 0.0012351081 | |  |
| anxiety | prescription_drugs | 0.0012211592 | | 0.0012211592 | |  |
| OGD_Q_1 | OGD_Q_11 | 0.0012173821 | | 0.0012173821 | |  |
| OGD_Q_3 | OGD_Q_11 | -0.0012007019 | | 0.0012007019 | |  |
| OGD_Q_1 | OGD_Q_7 | -0.0011322050 | | 0.0011322050 | |  |
| OGD_Q_2 | OGD_Q_10 | 0.0010877697 | | 0.0010877697 | |  |
| OGD_Q_4 | OGD_Q_7 | -0.0010740292 | | 0.0010740292 | |  |
| smoking_tobacco | Education | 0.0010389937 | | 0.0010389937 | |  |
| OGD_Q_8 | cannabis_drugs | -0.0010286940 | | 0.0010286940 | |  |
| smoking_tobacco | drinking_alcohol | -0.0009842536 | | 0.0009842536 | |  |
| depression | OGD_Q_10 | 0.0009263940 | | 0.0009263940 | |  |
| OGD_Q_8 | performance_drugs | -0.0008444467 | | 0.0008444467 | |  |
| OGD_Q_9 | non_cannabis_drugs | 0.0008395401 | | 0.0008395401 | |  |
| stress | OGD_Q_9 | -0.0007497085 | | 0.0007497085 | |  |
| depression | OGD_Q_7 | 0.0006686108 | | 0.0006686108 | |  |
| OGD_Q_1 | OGD_Q_3 | 0.0006012758 | | 0.0006012758 | |  |
| OGD_Q_7 | cannabis_drugs | -0.0005933266 | | 0.0005933266 | |  |
| depression | OGD_Q_4 | -0.0005547984 | | 0.0005547984 | |  |
| drinking_alcohol | Age | -0.0004985920 | | 0.0004985920 | |  |
| depression | cannabis_drugs | 0.0004787565 | | 0.0004787565 | |  |
| OGD_Q_4 | OGD_Q_8 | -0.0004757022 | | 0.0004757022 | |  |
| depression | OGD_Q_3 | -0.0002253218 | | 0.0002253218 | |  |
| OGD_Q_8 | drinking_alcohol | -0.0001861377 | | 0.0001861377 | |  |
| OGD_Q_1 | cannabis_drugs | 0.0001460013 | | 0.0001460013 | |  |
