# Supplementary material for: Gambling, trauma, and the mind: a network analysis of online gambling and personal well-being
Source: BMC Psychol. 2025 Nov 5;13:1226. doi: 10.1186/s40359-025-03516-z (PMC12587689; doi:10.1186/s40359-025-03516-z)
Supplement: Supplementary file 46 — Supplementary Material 46. [file 40359_2025_3516_MOESM46_ESM.docx]

# Ordered edge-weight differences (male adjusted networks)

| **Var1** | **Var2** | **Freq** | | **abs_diff** | |
| --- | --- | --- | --- | --- | --- |
| non_cannabis_drugs | prescription_drugs | -0.0298728908 | 0.0298728908 | |  |
| OGD_Q_9 | OGD_Q_11 | -0.0287268617 | 0.0287268617 | |  |
| Age | Education | -0.0285527806 | 0.0285527806 | |  |
| OGD_Q_10 | non_cannabis_drugs | -0.0269450756 | 0.0269450756 | |  |
| OGD_Q_6 | OGD_Q_9 | -0.0263149205 | 0.0263149205 | |  |
| OGD_Q_4 | OGD_Q_5 | -0.0253244922 | 0.0253244922 | |  |
| cannabis_drugs | non_cannabis_drugs | -0.0253159712 | 0.0253159712 | |  |
| OGD_Q_6 | OGD_Q_11 | -0.0240503674 | 0.0240503674 | |  |
| drinking_alcohol | cannabis_drugs | -0.0240198901 | 0.0240198901 | |  |
| OGD_Q_2 | OGD_Q_7 | 0.0236237953 | 0.0236237953 | |  |
| OGD_Q_1 | OGD_Q_10 | 0.0234001158 | 0.0234001158 | |  |
| non_cannabis_drugs | performance_drugs | -0.0218037271 | 0.0218037271 | |  |
| OGD_Q_2 | OGD_Q_4 | -0.0209050903 | 0.0209050903 | |  |
| OGD_Q_4 | OGD_Q_10 | -0.0205478820 | 0.0205478820 | |  |
| anxiety | Income | 0.0188080279 | 0.0188080279 | |  |
| OGD_Q_6 | OGD_Q_10 | 0.0184764615 | 0.0184764615 | |  |
| OGD_Q_2 | prescription_drugs | -0.0184220229 | 0.0184220229 | |  |
| OGD_Q_1 | OGD_Q_2 | -0.0184060854 | 0.0184060854 | |  |
| prescription_drugs | Age | -0.0164999676 | 0.0164999676 | |  |
| suicidal_ideation | Age | 0.0160792844 | 0.0160792844 | |  |
| OGD_Q_10 | cannabis_drugs | -0.0157714429 | 0.0157714429 | |  |
| OGD_Q_10 | performance_drugs | -0.0151715269 | 0.0151715269 | |  |
| OGD_Q_10 | OGD_Q_11 | 0.0142941172 | 0.0142941172 | |  |
| OGD_Q_1 | OGD_Q_4 | 0.0142438683 | 0.0142438683 | |  |
| OGD_Q_2 | non_cannabis_drugs | -0.0137435755 | 0.0137435755 | |  |
| OGD_Q_1 | OGD_Q_5 | 0.0130063490 | 0.0130063490 | |  |
| OGD_Q_4 | OGD_Q_6 | -0.0121878948 | 0.0121878948 | |  |
| stress | OGD_Q_2 | -0.0120940332 | 0.0120940332 | |  |
| smoking_tobacco | Education | 0.0117101437 | 0.0117101437 | |  |
| smoking_tobacco | drinking_alcohol | -0.0114360653 | 0.0114360653 | |  |
| anxiety | OGD_Q_1 | -0.0113662849 | 0.0113662849 | |  |
| OGD_Q_7 | OGD_Q_10 | -0.0113174157 | 0.0113174157 | |  |
| stress | suicidal_ideation | -0.0112980258 | 0.0112980258 | |  |
| OGD_Q_8 | Age | 0.0109768059 | 0.0109768059 | |  |
| OGD_Q_5 | OGD_Q_11 | 0.0107537870 | 0.0107537870 | |  |
| OGD_Q_2 | OGD_Q_5 | -0.0102717193 | 0.0102717193 | |  |
| OGD_Q_5 | OGD_Q_8 | -0.0102705642 | 0.0102705642 | |  |
| performance_drugs | Age | 0.0100027573 | 0.0100027573 | |  |
| depression | prescription_drugs | -0.0099296647 | 0.0099296647 | |  |
| OGD_Q_3 | OGD_Q_4 | -0.0099208517 | 0.0099208517 | |  |
| anxiety | stress | -0.0097629346 | 0.0097629346 | |  |
| stress | Age | 0.0096788328 | 0.0096788328 | |  |
| stress | Income | 0.0096406498 | 0.0096406498 | |  |
| OGD_Q_8 | OGD_Q_10 | -0.0095331367 | 0.0095331367 | |  |
| smoking_tobacco | cannabis_drugs | -0.0093842717 | 0.0093842717 | |  |
| stress | performance_drugs | -0.0090715979 | 0.0090715979 | |  |
| OGD_Q_10 | prescription_drugs | -0.0090244900 | 0.0090244900 | |  |
| depression | Income | 0.0088598597 | 0.0088598597 | |  |
| OGD_Q_1 | performance_drugs | -0.0083897561 | 0.0083897561 | |  |
| drinking_alcohol | Age | -0.0081753007 | 0.0081753007 | |  |
| depression | anxiety | -0.0080569915 | 0.0080569915 | |  |
| OGD_Q_5 | drinking_alcohol | -0.0076659625 | 0.0076659625 | |  |
| OGD_Q_3 | OGD_Q_10 | 0.0072274202 | 0.0072274202 | |  |
| OGD_Q_1 | cannabis_drugs | 0.0071623468 | 0.0071623468 | |  |
| anxiety | suicidal_ideation | -0.0071177185 | 0.0071177185 | |  |
| OGD_Q_4 | OGD_Q_9 | 0.0070084506 | 0.0070084506 | |  |
| OGD_Q_2 | OGD_Q_10 | 0.0069612944 | 0.0069612944 | |  |
| smoking_tobacco | performance_drugs | -0.0069144647 | 0.0069144647 | |  |
| OGD_Q_7 | Income | -0.0066992737 | 0.0066992737 | |  |
| depression | smoking_tobacco | -0.0066579337 | 0.0066579337 | |  |
| OGD_Q_4 | performance_drugs | 0.0066364725 | 0.0066364725 | |  |
| cannabis_drugs | prescription_drugs | -0.0065632406 | 0.0065632406 | |  |
| stress | OGD_Q_7 | 0.0064818732 | 0.0064818732 | |  |
| OGD_Q_3 | OGD_Q_7 | -0.0064219590 | 0.0064219590 | |  |
| OGD_Q_2 | OGD_Q_3 | 0.0062686897 | 0.0062686897 | |  |
| OGD_Q_7 | OGD_Q_9 | 0.0061431493 | 0.0061431493 | |  |
| anxiety | OGD_Q_4 | 0.0055370063 | 0.0055370063 | |  |
| OGD_Q_9 | non_cannabis_drugs | 0.0052438339 | 0.0052438339 | |  |
| OGD_Q_6 | OGD_Q_7 | 0.0051875410 | 0.0051875410 | |  |
| stress | OGD_Q_9 | -0.0049039601 | 0.0049039601 | |  |
| OGD_Q_2 | OGD_Q_6 | -0.0045149518 | 0.0045149518 | |  |
| anxiety | performance_drugs | -0.0044688264 | 0.0044688264 | |  |
| OGD_Q_8 | performance_drugs | -0.0042639704 | 0.0042639704 | |  |
| OGD_Q_4 | OGD_Q_7 | 0.0042471041 | 0.0042471041 | |  |
| OGD_Q_7 | OGD_Q_11 | -0.0041190948 | 0.0041190948 | |  |
| OGD_Q_8 | OGD_Q_11 | -0.0040791477 | 0.0040791477 | |  |
| depression | cannabis_drugs | -0.0040048589 | 0.0040048589 | |  |
| OGD_Q_2 | OGD_Q_9 | -0.0039898013 | 0.0039898013 | |  |
| cannabis_drugs | performance_drugs | -0.0038712507 | 0.0038712507 | |  |
| OGD_Q_3 | OGD_Q_11 | -0.0037536946 | 0.0037536946 | |  |
| anxiety | cannabis_drugs | 0.0035589191 | 0.0035589191 | |  |
| OGD_Q_1 | Age | 0.0035563075 | 0.0035563075 | |  |
| depression | suicidal_ideation | -0.0035342690 | 0.0035342690 | |  |
| OGD_Q_4 | OGD_Q_11 | 0.0034925910 | 0.0034925910 | |  |
| OGD_Q_6 | OGD_Q_8 | 0.0033989528 | 0.0033989528 | |  |
| anxiety | OGD_Q_9 | -0.0030947819 | 0.0030947819 | |  |
| OGD_Q_2 | OGD_Q_11 | -0.0030706045 | 0.0030706045 | |  |
| OGD_Q_1 | OGD_Q_8 | -0.0029626544 | 0.0029626544 | |  |
| cannabis_drugs | Education | 0.0027176565 | 0.0027176565 | |  |
| OGD_Q_9 | OGD_Q_10 | -0.0025975292 | 0.0025975292 | |  |
| OGD_Q_5 | OGD_Q_6 | -0.0025650025 | 0.0025650025 | |  |
| smoking_tobacco | non_cannabis_drugs | -0.0024988039 | 0.0024988039 | |  |
| OGD_Q_1 | OGD_Q_3 | -0.0024971247 | 0.0024971247 | |  |
| depression | OGD_Q_4 | -0.0024884310 | 0.0024884310 | |  |
| OGD_Q_3 | OGD_Q_8 | 0.0024693866 | 0.0024693866 | |  |
| OGD_Q_3 | OGD_Q_9 | -0.0021611723 | 0.0021611723 | |  |
| depression | OGD_Q_7 | -0.0020670634 | 0.0020670634 | |  |
| OGD_Q_5 | performance_drugs | 0.0019430738 | 0.0019430738 | |  |
| OGD_Q_3 | OGD_Q_5 | -0.0018067755 | 0.0018067755 | |  |
| suicidal_ideation | OGD_Q_6 | -0.0017129598 | 0.0017129598 | |  |
| OGD_Q_5 | OGD_Q_7 | -0.0013471670 | 0.0013471670 | |  |
| OGD_Q_1 | OGD_Q_6 | -0.0013082392 | 0.0013082392 | |  |
| anxiety | Age | 0.0010871226 | 0.0010871226 | |  |
| depression | OGD_Q_1 | -0.0010409656 | 0.0010409656 | |  |
| depression | stress | -0.0007389322 | 0.0007389322 | |  |
| suicidal_ideation | Income | 0.0006503140 | 0.0006503140 | |  |
| OGD_Q_1 | OGD_Q_7 | -0.0005115186 | 0.0005115186 | |  |
| OGD_Q_4 | OGD_Q_8 | -0.0001601314 | 0.0001601314 | |  |
