# Supplementary material for: Gambling, trauma, and the mind: a network analysis of online gambling and personal well-being
Source: BMC Psychol. 2025 Nov 5;13:1226. doi: 10.1186/s40359-025-03516-z (PMC12587689; doi:10.1186/s40359-025-03516-z)
Supplement: Supplementary file 47 — Supplementary Material 47. [file 40359_2025_3516_MOESM47_ESM.docx]

# Ordered edge-weight differences (female adjusted networks)

| **Var1** | **Var2** | | **Freq** | | **abs_diff** | |
| --- | --- | --- | --- | --- | --- | --- |
| OGD_Q_6 | non_cannabis_drugs | 0.0285181158 | | 0.0285181158 | |  |
| non_cannabis_drugs | performance_drugs | -0.0275656788 | | 0.0275656788 | |  |
| OGD_Q_1 | OGD_Q_8 | -0.0235565237 | | 0.0235565237 | |  |
| cannabis_drugs | performance_drugs | 0.0167545839 | | 0.0167545839 | |  |
| OGD_Q_6 | OGD_Q_8 | 0.0167328943 | | 0.0167328943 | |  |
| smoking_tobacco | performance_drugs | -0.0145604775 | | 0.0145604775 | |  |
| prescription_drugs | performance_drugs | -0.0144573392 | | 0.0144573392 | |  |
| anxiety | Income | 0.0143542094 | | 0.0143542094 | |  |
| OGD_Q_6 | OGD_Q_9 | 0.0128487994 | | 0.0128487994 | |  |
| cannabis_drugs | prescription_drugs | -0.0123364451 | | 0.0123364451 | |  |
| OGD_Q_8 | Income | -0.0121108572 | | 0.0121108572 | |  |
| suicidal_ideation | Age | -0.0119240619 | | 0.0119240619 | |  |
| OGD_Q_8 | cannabis_drugs | -0.0118883227 | | 0.0118883227 | |  |
| depression | Income | 0.0115297466 | | 0.0115297466 | |  |
| anxiety | stress | -0.0110586830 | | 0.0110586830 | |  |
| OGD_Q_5 | OGD_Q_8 | 0.0110392497 | | 0.0110392497 | |  |
| stress | suicidal_ideation | -0.0109021404 | | 0.0109021404 | |  |
| OGD_Q_7 | non_cannabis_drugs | -0.0106169644 | | 0.0106169644 | |  |
| OGD_Q_9 | OGD_Q_10 | -0.0105403777 | | 0.0105403777 | |  |
| OGD_Q_6 | OGD_Q_7 | 0.0100649995 | | 0.0100649995 | |  |
| anxiety | suicidal_ideation | -0.0100371488 | | 0.0100371488 | |  |
| OGD_Q_7 | OGD_Q_9 | -0.0096355310 | | 0.0096355310 | |  |
| OGD_Q_6 | OGD_Q_10 | 0.0094138539 | | 0.0094138539 | |  |
| anxiety | smoking_tobacco | -0.0089077258 | | 0.0089077258 | |  |
| suicidal_ideation | smoking_tobacco | -0.0088299156 | | 0.0088299156 | |  |
| OGD_Q_1 | performance_drugs | -0.0087187043 | | 0.0087187043 | |  |
| OGD_Q_9 | OGD_Q_11 | -0.0084397249 | | 0.0084397249 | |  |
| OGD_Q_7 | OGD_Q_10 | -0.0082889990 | | 0.0082889990 | |  |
| OGD_Q_6 | OGD_Q_11 | 0.0082708696 | | 0.0082708696 | |  |
| OGD_Q_3 | OGD_Q_4 | 0.0082496242 | | 0.0082496242 | |  |
| OGD_Q_2 | cannabis_drugs | -0.0081870611 | | 0.0081870611 | |  |
| cannabis_drugs | non_cannabis_drugs | -0.0081836565 | | 0.0081836565 | |  |
| depression | stress | -0.0081807880 | | 0.0081807880 | |  |
| OGD_Q_5 | OGD_Q_10 | -0.0081370216 | | 0.0081370216 | |  |
| OGD_Q_7 | OGD_Q_11 | -0.0074180634 | | 0.0074180634 | |  |
| drinking_alcohol | non_cannabis_drugs | -0.0074094379 | | 0.0074094379 | |  |
| Age | Education | -0.0073751903 | | 0.0073751903 | |  |
| OGD_Q_2 | OGD_Q_9 | -0.0072614843 | | 0.0072614843 | |  |
| OGD_Q_10 | OGD_Q_11 | -0.0072207730 | | 0.0072207730 | |  |
| stress | Age | -0.0071415825 | | 0.0071415825 | |  |
| OGD_Q_2 | OGD_Q_6 | 0.0070321671 | | 0.0070321671 | |  |
| OGD_Q_5 | OGD_Q_9 | -0.0070138509 | | 0.0070138509 | |  |
| stress | smoking_tobacco | -0.0068689185 | | 0.0068689185 | |  |
| OGD_Q_2 | OGD_Q_10 | -0.0066820344 | | 0.0066820344 | |  |
| OGD_Q_2 | non_cannabis_drugs | -0.0064992911 | | 0.0064992911 | |  |
| OGD_Q_2 | OGD_Q_11 | -0.0062134964 | | 0.0062134964 | |  |
| OGD_Q_2 | OGD_Q_7 | -0.0059902478 | | 0.0059902478 | |  |
| depression | OGD_Q_1 | 0.0059571737 | | 0.0059571737 | |  |
| OGD_Q_5 | OGD_Q_7 | -0.0058226077 | | 0.0058226077 | |  |
| OGD_Q_1 | OGD_Q_5 | 0.0057864617 | | 0.0057864617 | |  |
| OGD_Q_2 | OGD_Q_5 | -0.0057001150 | | 0.0057001150 | |  |
| OGD_Q_6 | drinking_alcohol | 0.0054260807 | | 0.0054260807 | |  |
| OGD_Q_3 | prescription_drugs | -0.0053278941 | | 0.0053278941 | |  |
| OGD_Q_5 | non_cannabis_drugs | -0.0051146545 | | 0.0051146545 | |  |
| OGD_Q_7 | prescription_drugs | -0.0050344379 | | 0.0050344379 | |  |
| OGD_Q_4 | OGD_Q_5 | -0.0049801337 | | 0.0049801337 | |  |
| OGD_Q_4 | OGD_Q_9 | -0.0049609336 | | 0.0049609336 | |  |
| OGD_Q_1 | OGD_Q_10 | 0.0048274210 | | 0.0048274210 | |  |
| Age | Income | 0.0047325802 | | 0.0047325802 | |  |
| OGD_Q_1 | OGD_Q_6 | 0.0046434566 | | 0.0046434566 | |  |
| OGD_Q_3 | OGD_Q_5 | 0.0045074007 | | 0.0045074007 | |  |
| OGD_Q_3 | OGD_Q_9 | 0.0044938798 | | 0.0044938798 | |  |
| OGD_Q_4 | OGD_Q_7 | -0.0044552065 | | 0.0044552065 | |  |
| depression | anxiety | -0.0042757382 | | 0.0042757382 | |  |
| depression | smoking_tobacco | -0.0042340061 | | 0.0042340061 | |  |
| OGD_Q_8 | OGD_Q_10 | 0.0039283700 | | 0.0039283700 | |  |
| prescription_drugs | Age | 0.0039092567 | | 0.0039092567 | |  |
| OGD_Q_3 | OGD_Q_7 | 0.0038874828 | | 0.0038874828 | |  |
| OGD_Q_2 | OGD_Q_4 | -0.0037040037 | | 0.0037040037 | |  |
| smoking_tobacco | cannabis_drugs | -0.0035963708 | | 0.0035963708 | |  |
| OGD_Q_4 | OGD_Q_11 | -0.0035585126 | | 0.0035585126 | |  |
| OGD_Q_4 | OGD_Q_10 | -0.0034540420 | | 0.0034540420 | |  |
| smoking_tobacco | drinking_alcohol | -0.0033869962 | | 0.0033869962 | |  |
| depression | suicidal_ideation | -0.0033369141 | | 0.0033369141 | |  |
| smoking_tobacco | non_cannabis_drugs | 0.0032528578 | | 0.0032528578 | |  |
| OGD_Q_8 | OGD_Q_11 | -0.0030874621 | | 0.0030874621 | |  |
| anxiety | Age | 0.0028349077 | | 0.0028349077 | |  |
| anxiety | drinking_alcohol | -0.0026700673 | | 0.0026700673 | |  |
| OGD_Q_2 | OGD_Q_3 | 0.0026232091 | | 0.0026232091 | |  |
| Education | Income | -0.0026200116 | | 0.0026200116 | |  |
| smoking_tobacco | Age | 0.0025959004 | | 0.0025959004 | |  |
| OGD_Q_4 | OGD_Q_6 | 0.0025373825 | | 0.0025373825 | |  |
| OGD_Q_1 | OGD_Q_2 | 0.0025320199 | | 0.0025320199 | |  |
| OGD_Q_8 | OGD_Q_9 | -0.0024438216 | | 0.0024438216 | |  |
| OGD_Q_7 | OGD_Q_8 | -0.0024406897 | | 0.0024406897 | |  |
| depression | OGD_Q_6 | -0.0023666131 | | 0.0023666131 | |  |
| depression | cannabis_drugs | 0.0016621638 | | 0.0016621638 | |  |
| anxiety | prescription_drugs | 0.0015199290 | | 0.0015199290 | |  |
| OGD_Q_1 | OGD_Q_4 | 0.0014413898 | | 0.0014413898 | |  |
| depression | OGD_Q_3 | 0.0014411653 | | 0.0014411653 | |  |
| smoking_tobacco | Education | -0.0014124495 | | 0.0014124495 | |  |
| OGD_Q_1 | OGD_Q_11 | 0.0013683374 | | 0.0013683374 | |  |
| OGD_Q_5 | OGD_Q_6 | -0.0013608253 | | 0.0013608253 | |  |
| drinking_alcohol | Income | -0.0012127439 | | 0.0012127439 | |  |
| stress | OGD_Q_11 | 0.0011497473 | | 0.0011497473 | |  |
| OGD_Q_3 | OGD_Q_10 | 0.0011150783 | | 0.0011150783 | |  |
| OGD_Q_1 | OGD_Q_7 | 0.0010691989 | | 0.0010691989 | |  |
| OGD_Q_3 | non_cannabis_drugs | -0.0010109203 | | 0.0010109203 | |  |
| OGD_Q_2 | OGD_Q_8 | 0.0009347351 | | 0.0009347351 | |  |
| OGD_Q_1 | smoking_tobacco | -0.0008088775 | | 0.0008088775 | |  |
| OGD_Q_3 | OGD_Q_11 | 0.0006427012 | | 0.0006427012 | |  |
| OGD_Q_7 | performance_drugs | 0.0005416008 | | 0.0005416008 | |  |
| stress | OGD_Q_1 | 0.0005176231 | | 0.0005176231 | |  |
| depression | OGD_Q_2 | 0.0005095623 | | 0.0005095623 | |  |
| OGD_Q_1 | prescription_drugs | 0.0004914118 | | 0.0004914118 | |  |
| depression | OGD_Q_10 | -0.0004426503 | | 0.0004426503 | |  |
| OGD_Q_4 | drinking_alcohol | 0.0003990835 | | 0.0003990835 | |  |
| drinking_alcohol | cannabis_drugs | 0.0003973633 | | 0.0003973633 | |  |
| OGD_Q_6 | prescription_drugs | -0.0002637799 | | 0.0002637799 | |  |
| depression | OGD_Q_9 | 0.0001709821 | | 0.0001709821 | |  |
